# Supplementary material for: Movement side effects of antipsychotic drugs in adults with and without intellectual disability: UK population-based cohort study
Source: BMJ Open. 2017 Aug 3;7(8):e017406. doi: 10.1136/bmjopen-2017-017406 (PMC5724123; doi:10.1136/bmjopen-2017-017406)
Supplement: Supplementary file 1 [file bmjopen-2017-017406supp001.docx]

| **Antipsychotic movement side-effects by type** | |
| --- | --- |
|  |  |
| **Acute dystonia** | |
| 16A3.00 | Wry neck/torticollis |
| 16A3.11 | Torticollis - symptom |
| 1B25.00 | Has "spasms" |
| 1B25.11 | Spasms - symptom |
| 1B35.00 | Attacks of rigidity |
| 1B36.00 | Trismus present |
| 22B2.00 | O/E - carpopedal spasm |
| 2942000 | Trismus |
| 2974.00 | O/E - spasm/tic |
| 2974.11 | O/E - spasm |
| 7Q04000 | Torsion dystonias other involuntary movements drugs band 1 |
| F137.00 | Symptomatic torsion dystonia |
| F137200 | Drug-induced dystonia |
| F137y00 | Other specified symptomatic torsion dystonia |
| F137z00 | Symptomatic torsion dystonia NOS |
| F138.00 | Fragments of torsion dystonia |
| F138000 | Blepharospasm |
| F138200 | Spasmodic torticollis |
| F13X.00 | Dystonia, unspecified |
| F4Jy911 | Oculogyric crisis |
| Fyu2400 | [X]Other dystonia |
| Fyu2A00 | [X]Dystonia, unspecified |
| N135.00 | Torticollis unspecified |
| N135000 | Intermittent torticollis |
| N135z00 | Torticollis NOS |
| R010200 | [D]Spasms NOS |
| R010600 | [D] Trismus |
| R017000 | [D]Carpopedal spasm |
|  |  |
| **Pseudo-Parkinsonism** | |
| 1B22.00 | Has a tremor |
| 1B22.11 | Tremor symptom |
| 1B22.12 | Shaking |
| 1B23.00 | Trembles |
| 1B23.11 | Trembles - symptom |
| 294..11 | O/E - rigid muscle |
| 2942.00 | O/E - muscle tone hypertonic |
| 2944.00 | O/E - muscle rigid - cogwheel |
| 2944.11 | O/E - cog wheel rigidity |
| 297A.00 | O/E - Parkinsonian tremor |
| 2987.00 | O/E -Parkinson flexion posture |
| 2987.11 | O/E - Parkinson posture |
| 2994.00 | O/E-festination-Parkinson gait |
| 2994.11 | O/E - Parkinson gait |
| F121.00 | Parkinsonism secondary to drugs |
| F121.11 | Drug induced parkinsonism |
| F12W.00 | Secondary parkinsonism due to other external agents |
| F12X.00 | Secondary parkinsonism, unspecified |
| F131200 | Drug-induced tremor |
| Fyu2000 | [X]Other drug-induced secondary parkinsonism |
| Fyu2100 | [X]Other secondary parkinsonism |
| Fyu2900 | [X]Secondary parkinsonism, unspecified |
| R010300 | [D]Tremor NOS |
| Fyu2B00 | [X]Secondary parkinsonism due to other external agents |
|  |  |
| **Akathisia** | |
| 1B1O.00 | Restless |
| 1P04.00 | C/O - akathisia |
|  |  |
| **Tardive dyskinesia** | |
| F138100 | Orofacial dyskinesia |
| F138111 | Tardive dyskinesia |
| 297..00 | O/E - involuntary movements |
| 297Z.00 | O/E - involuntary movement NOS |
| 1B2..00 | Involuntary movement symptom |
| 1B2Z.00 | Involuntary movemt.symptom NOS |
| R010.00 | [D]Abnormal involuntary movements |
| R010z00 | [D]Abnormal involuntary movement NOS |
| Ryu3000 | [X]Other and unspecified abnormal involuntary movements |
| 1B2Z.00 | Involuntary movemt.symptom NOS |
| 1B2..00 | Involuntary movement symptom |
|  |  |
| **Other/misc.** | |
| Fyu2700 | [X]Other specified extrapyramidal and movement disorders |
| R013.11 | [D]Dyskinesia |
| F13z.00 | Other/unspecified extrapyramidal/abnormal movement disorders |
| F13z000 | Unspecified extrapyramidal disease |
| F13zz00 | Extrapyramidal disease and abnormal movement disorder NOS |
| Fyu2.00 | [X]Extrapyramidal and movement disorders |
| 29M..00 | Extrapyramidal movements |
| F13..00 | Other extrapyramidal disease and abnormal movement disorders |
| F13..11 | Extrapyramidal disease excluding Parkinson's disease |
| ZS42500 | Extrapyramidal dysarthria |
|  |  |
| **Neuroleptic malignant syndrome** | |
| F122.00 | Malignant neuroleptic syndrome |
