# Supplementary material for: Movement side effects of antipsychotic drugs in adults with and without intellectual disability: UK population-based cohort study
Source: BMJ Open. 2017 Aug 3;7(8):e017406. doi: 10.1136/bmjopen-2017-017406 (PMC5724123; doi:10.1136/bmjopen-2017-017406)
Supplement: Author's manuscript [file bmjopen-2017-017406.draft_revisions.pdf]

BMJ Open

Movement side-effects of antipsychotic drugs in adults with intellectual disability: cohort study

Journal:	*BMJ Open*
Manuscript ID	bmjopen-2017-017406
Article Type:	Research
Date Submitted by the Author:	24-Apr-2017
Complete List of Authors:	Sheehan, Rory; University College London, Division of Psychiatry<br>Horsfall, Laura; University College London, Research Department of Primary Care and Population Health<br>Strydom, Andre; Division of Psychiatry<br>Osborn, David; University College London , Division of Psychiatry<br>Walters, Kate; University College London, Research Department of Primary Care and Population Health<br>HASSIOTIS, ANGELA; University College London, Division of Psychiatry
**Primary Subject Heading**:	Mental health
Secondary Subject Heading:	Pharmacology and therapeutics
Keywords:	MENTAL HEALTH, PSYCHIATRY, Adverse events < THERAPEUTICS

SCHOLARONE™ Manuscripts

For peer review only

TITLE PAGE

Movement side-effects of antipsychotic drugs in adults with intellectual disability: cohort study

Dr Rory Sheehan*, MSc, Division of Psychiatry, University College London

Dr Laura Horsfall*, PhD, Research Department of Primary Care and Population Health, University College London

Dr André Strydom, PhD, Division of Psychiatry, University College London

Professor David Osborn, PhD, Division of Psychiatry, University College London

Dr Kate Walters, PhD, Research Department of Primary Care and Population Health, University College London

Professor Angela Hassiotis, PhD, Division of Psychiatry, University College London

*Rory Sheehan and Laura Horsfall contributed equally to this work.

Corresponding author: Dr Rory Sheehan

Address: Division of Psychiatry, University College London, 6th Floor Maple House, 149 Tottenham Court Road, London W1T 7NF

Telephone: 0207-679-9201

E-mail: r.sheehan@ucl.ac.uk

For peer review only

ABSTRACT

Movement side-effects of antipsychotic drugs in adults with intellectual disability: cohort study

Objectives To measure the incidence of movement side-effects of antipsychotic drugs in adults with intellectual disability and compare rates with adults without intellectual disability.

Design Cohort study using data from The Health Improvement Network

Setting Primary care

Participants Adults with intellectual disability prescribed antipsychotic drugs matched to a control group of adults without intellectual disability prescribed antipsychotic drugs.

Outcome measures New records of movement side-effect including, acute dystonias, akathisia, pseudo-Parkinsonism, tardive dyskinesia, and neuroleptic malignant syndrome.

Results 9,013 adults with intellectual disability and a control cohort of 34,242 adults without intellectual disability together contributed 148,709 person-years data. The overall incidence of recorded movement side-effects was 275 per 10,000 person-years (95% confidence interval 256-296) in the intellectual disability group and 248 per 10,000 person-years (95% confidence interval 237-260) in the control group. The incidence of any recorded movement side-effect was significantly greater in people with intellectual disability compared to those without (incidence rate ratio 1.30, 95% confidence interval 1.18 to 1.42, p<0.001, after adjustment for potential confounders), with pseudo-Parkinsonism and akathisia showing the greatest difference between the groups. Neuroleptic malignant syndrome, although occurring infrequently, was three times more common in people with intellectual disability prescribed antipsychotic drugs (incidence rate ratio 3.03, 95% confidence interval 1.26 to 7.30, p=0.013). Differences in rates of movement side-effects between the groups were not

For peer review only

due to differences in the proportions prescribed first- and second-generation antipsychotic drugs.

Conclusions This study provides evidence to substantiate the long-held assumption that people with intellectual disability are more susceptible to movement side-effects of antipsychotic drugs. Assessment for movement side-effects should be integral to antipsychotic drug monitoring in people with intellectual disability. Regular medication review is essential to ensure optimal prescribing in this group.

For peer review only

ARTICLE SUMMARY

Strengths and limitations of this study

- This study includes a very large number of people with and without intellectual disability who have been prescribed antipsychotic drugs.
- The Health Improvement Network is a UK primary care database that contains accurate recording of demographic and clinical information and drug prescribing.
- This is the first study to directly compare the rates of movement side-effects of antipsychotic drugs between people with intellectual disability and those without, and offers new insights into the risk-benefit ratio of antipsychotic drug prescribing to people with intellectual disability.
- Recording of movement side-effects of antipsychotic drugs in primary care has not been validated.
- Antipsychotic drugs prescribed outside primary care and movement side-effects identified in other settings may not have been recorded by our method.

For peer review only

FUNDING STATEMENT

This work was supported by The Baily Thomas Charitable Fund grant number 518669 and the National Institute for Health Research grant reference RP-DG-0611-10003. The funders had no role in study design, data collection and analysis, decision to publish, or preparation of the manuscript.

COMPETING INTERESTS

Rory Sheehan reports no conflicts of interest.

Laura Horsfall reports no conflicts of interest.

André Strydom reports grants from Roche pharmaceuticals, outside the submitted work.

David Osborn reports no conflicts of interest.

Kate Walters reports no conflicts of interest.

Angela Hassiotis reports no conflicts of interest.

AUTHOR CONTRIBUTIONS

Rory Sheehan developed the idea and method for the study, interpreted the results, and wrote the manuscript. Rory Sheehan is guarantor.

Laura Horsfall developed the idea and method for the study, performed the data extraction and analysis, interpreted the results, and wrote the manuscript.

André Strydom developed the idea and method for the study, interpreted the results, and wrote the manuscript.

David Osborn developed the idea and method for the study, interpreted the results, and wrote the manuscript.

Kate Walters developed the idea and method for the study, interpreted the results, and wrote the manuscript.

Angela Hassiotis developed the idea and method for the study, interpreted the results, and wrote the manuscript.

DATA SHARING STATEMENT

Copies of Read code lists used in this study are available from the authors, on request.

For peer review only

MANUSCRIPT

Movement side-effects of antipsychotic drugs in adults with intellectual disability: cohort study

Introduction

Movement (extra-pyramidal) side-effects, including acute dystonias, akathisia, Parkinsonism, and tardive dyskinesia, are a well-recognised complication of antipsychotic drugs which are thought to occur secondary to antagonism of dopamine D2 receptors in the striatum and meso-cortex.[1] Movement side-effects can be distressing, disabling, and difficult to treat and their presence is associated with poor medication compliance, stigma, and reduced quality of life.[2]

Intellectual disability (ID) is a lifelong condition characterised by global deficits in cognitive and adaptive functioning. People with ID experience relatively high rates of mental illness[3] and many worldwide and in the UK are prescribed antipsychotic drugs. There has been renewed focus on the appropriateness of antipsychotic drug prescribing in people with ID following recent evidence that antipsychotic drugs are often used in the absence of an underlying diagnosed mental illness,[4] in many cases in an attempt to manage challenging behaviour, despite a lack of evidence that they are effective in this context.[5] There has been relatively little formal investigation of antipsychotic drug side-effects in people with ID and most of our knowledge is extrapolated from studies conducted in people of average intelligence. People with ID are often considered to be at greater risk of antipsychotic drug-induced movement side-effects than people without ID[6] but no studies directly compare rates between the two groups. Furthermore, knowledge of a specific mechanism that might underpin any association between ID and movement side-effects extends only to a vague theory that organic brain dysfunction makes centrally-mediated side-effects of psychotropic drugs more likely.[7]

For peer review only

We undertook a cohort study using a large nationally-representative database to compare the incidence of recorded movement side-effects in adults with and without ID who are prescribed antipsychotic drugs.

Methods

Data source

Data were obtained from The Health Improvement Network (THIN), a large UK primary care database that contains the electronic health records of more than 3.7 million active patients in over 550 general practices (http://www.epic-uk.org/our-data/our-data.shtml). The patients included in the database are representative of the UK population in age, sex, and morbidity and mortality.[8,9] The THIN database contains records of symptoms, signs, diagnoses, and treatments; data is added by general practitioners (primary care physicians) using a standardised clinical dictionary of Read codes.[10] Recording of illness in primary care records has been shown to be accurate and all prescribed medication must be issued through the system. The primary care record therefore is a suitable means of conducting pharmaco-epidemiological research.

THIN data are pseudonymised at source and made available to researchers who have purchased a license. THIN has overall ethical approval to collate data and this study received approval from the THIN Scientific Review Committee (reference 14-071).

Study cohort

For this study, all adults with recorded ID and a history of oral antipsychotic drug prescription were extracted using a previously-defined and tested list of diagnostic Read codes (including codes for ID and conditions associated with ID) and antipsychotic drug codes,[4] based on chapters of the *British National Formulary*. General practitioners are incentivised by the Quality Outcomes Framework (QOF) to keep a register of people with ID which improves recording in the database. The study period was 1st January 1999 to 31st December 2014. Entry to the cohort was set as the date of the first antipsychotic drug

For peer review only

prescription issued after the latest of; registration with the GP practice contributing data; the patient's 18th birthday; the start of the study period; the date the practice achieved compliance with standard measures of data quality.[11,12] People contributed person-years (PYs) of data from entry to cohort exit. Exit from the cohort was defined as the first of; the final antipsychotic drug prescription plus the length of the prescription; de-registration with the GP practice contributing data; the end of the study period; or death. People also exited at the date they developed a movement side-effect as they were no longer considered at risk after this time.

A comparison cohort of people prescribed antipsychotic drugs but without ID was extracted using stratified sampling within each GP practice with frequency matching to ensure similar population-level characteristics across the two cohorts in terms of age, gender, and year of antipsychotic prescription. Up to six people without ID were selected for every person with ID and the same criteria were used to define cohort entry and exit.

A Read code list for movement side-effects was developed using previously-described methodology and applied to the cohort to determine incidence of movement side-effects[13] (list available from the authors, on request). Movement side-effects were categorised as; acute dystonias; akathisia; pseudo-Parkinsonism; or tardive dyskinesia; in accordance with orthodox classification. A separate category was established for neuroleptic malignant syndrome (NMS), being a very specific adverse effect, and a further category included for broad codes which could not be sub-categorised. Prescriptions for selected antimuscarinic drugs (procyclidine, orphenadrine, trihexyphenidyl) were used as proxy indicators of movement side-effects in those prescribed antipsychotic drugs. People were defined as having a history of movement disorder if a relevant Read code was applied (or antimuscarinic drug prescribed) prior to cohort entry or within six months of registration with the practice, as this has been shown to improve the validity of incidence calculations.[14]

Covariates

Sociodemographic covariates included age, sex, calendar year, and the Townsend Deprivation Score (a composite score in fifths based on postcode and Census recording of

For peer review only

local unemployment, car ownership, home ownership, and overcrowding).[15] Other covariates included history of antipsychotic use at cohort entry, antipsychotic average daily dose, and days on treatment between the start and end of follow-up. Average daily dose was measured as Chlorpromazine equivalents (CPZE) to account for polypharmacy and those who switched drugs during follow-up. Where we were unable to extract daily dose data (for example, in the minority of cases where the duration of a prescription was not recorded) the prescribed dose for the previous or subsequent prescription for the same drug and formulation was used. It was not possible to estimate the daily dose for 5% of prescriptions and these were excluded from the study. Prescriptions for drug doses above three times the upper licensed limit were excluded as probable coding errors (<1%).

Statistical analysis

Multivariable mixed Poisson regression was used to calculate incident rates of movement disorders during exposure to antipsychotic drugs in people with and without ID by calendar year, adjusted for any temporal changes in age and sex. General practice was included as a random effect to account for any data clustering. Calendar year was initially modelled as a continuous variable and we then used the likelihood ratio test to compare this with a model in which year was entered as a categorical variable to examine the possibility of non-linear time trends.

Multivariable mixed Poisson regression was also used to compare the rates of movement disorder during exposure to antipsychotic drugs in people with and without ID, adjusted for covariates. We conducted a sensitivity analysis where we restricted the analysis to time periods when people were exclusively prescribed first- or second-generation antipsychotic drugs, and further when we restricted the analysis to times when only risperidone was prescribed. All analyses were repeated after excluding people with a diagnosis of idiopathic Parkinson's disease.

We considered a p-value of 0.05 to be statistically significant (two-tailed) and used Stata version 13 for all analyses (StataCorp, TX).

For peer review only

Results

In total 9,039 people with ID met inclusion criteria and were matched to 34,242 people without ID, and together contributed 148,709 person-years (PYs) data. The two cohorts were similar in terms of age, sex, level of social deprivation, and history of movement disorder at cohort entry (table 1). The prevalence of movement disorder at baseline was slightly higher for people with ID and a history of antipsychotic use (31%) compared to those without ID and a history of antipsychotic drug use (24%) but the proportions of those with a history of movement disorder without antipsychotic drug use were equal (6%) at cohort entry. Average daily dose of antipsychotic was similar between the two groups but those with ID had longer time periods between their first and last antipsychotic prescription and more days on treatment between those dates. Table 2 shows the distribution of antipsychotic drugs prescribed to the study cohort. Risperidone was the most common drug prescribed in the ID cohort (28.5% prescriptions to people with ID, 14.7% prescriptions in the non-ID cohort); other drugs were prescribed in roughly equal proportions between the two groups.

[Table 1 near here]

[Table 2 near here]

The overall incidence of recorded movement side-effects was 275 per 10,000PYs (95% confidence interval (CI) 256-296) in the ID group and 248 per 10,000PYs (95%CI 237-260) in the non-ID group (table 3). Pseudo-Parkinsonism was the most commonly recorded movement side-effect in both groups. After adjustment, the incidence rate of any movement disorder was 30% higher in people with ID compared to those without ID (IRR 1.30, 95%CI 1.18 to 1.42, $p<0.001$). Similar differences in movement side-effect recording were noted when defined by diagnostic Read codes or by proxy, using prescription for antimuscarinic drugs. The incidence rates of akathisia, pseudo-Parkinsonism, and neuroleptic malignant syndrome were significantly higher in those with ID compared with those without ID.

For peer review only

Analysis restricted to periods when people were exclusively prescribed first- or second-generation antipsychotic drugs did not change the results; movement side-effects were still significantly more likely to be recorded in people with ID compared to those without (table 4). Excluding people with Parkinson's disease from the analysis (n=451) had no meaningful effect on any of the results.

[Table 3 near here]

[Table 4 near here]

Time trend analysis showed that the incidence of recording of movement side-effects in those prescribed antipsychotic drugs fell significantly over the course of the study period in both groups (figure 1); each calendar year was associated with a 5% decline in the recording of movement side-effects in people with ID (95%CI 2-8%, $p<0.0001$) and a 7% decline in people without ID (95%CI 5-9%, $p<0.001$), after accounting for changes in cohort age and sex. Prescriptions for antimuscarinic drugs declined by 3% per year in people with ID (95%CI 1-5%, $p=0.002$) and 5% per year in people without ID (95%CI 4-7%, $p<0.001$). Average daily antipsychotic dose, measured in Chlorpromazine equivalents, remained broadly constant during the study period (data not shown).

[Figure 1 near here]

Discussion

People with ID have long been considered at greater risk of adverse side-effects of antipsychotic drugs. However to date very little evidence has been presented to substantiate this belief. Our data suggest that people with ID are more likely to experience movement side-effects of antipsychotic drugs; this finding is robust and persists when movement side-effects are defined by diagnostic Read code alone and when they are measured using prescription of anti-muscarinic drugs as proxy.

For peer review only

Most people in our cohort received a second-generation antipsychotic drug. The types of drugs that were prescribed in our study were broadly similar between the group with ID and the group without, and were consistent with other recent data examining antipsychotic prescribing in community-dwelling adults living in the UK.[16] The exception was risperidone, which was prescribed more frequently to people with ID; this accords with other studies[17] and is likely to reflect an attempt by clinicians to prescribe the antipsychotic with greatest (albeit still limited) evidence of benefit for challenging behaviour in people with ID.[5,18] The difference in incidence of movement side-effects between people with and without ID remained when first- and second-generation agents were considered separately, and when risperidone was considered alone, suggesting that headline differences between the groups were not due to different prescribing practices.

Although their improved movement side-effect profile has been considered a major advantage of second-generation antipsychotic drugs, more recent evaluation of the evidence suggests that the initial enthusiasm for second-generation agents was misplaced and largely based on studies that made unequal comparisons between second-generation and high-potency first-generation drugs.[19,20] In this study we did not set out to compare movement side-effects between first and second-generation agents but we observed that the prescription of second-generation agents was associated with a slightly lower incidence of recorded movement side-effects in people with and without ID; clearly further work is needed to provide definitive data on this contentious aspect of antipsychotic drug side-effects.

There is a lack of work with which we can directly compare our results; previous studies that investigate movement disorders in people with ID who take anti-psychotic drugs have used differing methods to define and ascertain movement disorder, selected particular populations (often convenience sampling those residing in institutions), and tend to report point prevalence figures. None have directly compared rates of movement side-effect in people with ID to controls without ID. Nevertheless, it is clear that antipsychotic drug-induced movement side-effects are reasonably common in people with ID. In once recent study of hospitalised patients with borderline-mild ID and challenging behaviour, almost half were found to have a movement disorder, and the presence of movement disorder was

For peer review only

more likely in those prescribed antipsychotic medication.[21] The most common type was Parkinsonism, as in our study. De Kuijper and colleagues in the Netherlands report that just over half of their sample with ID who had been taking antipsychotic drugs for more than a year had evidence of movement side-effects.[22]

It is interesting that in our study the rates of recording of tardive dyskinesia were relatively low and it was not more frequently recorded in people with ID. Previous work has shown tardive dyskinesia to be common; one study of institutionalised adults with ID taking long-term antipsychotic drugs found a prevalence rate of tardive dyskinesia of 45%.[23] Spontaneous dyskinesias are also common in people with ID[24] and it is possible that drug-induced tardive dyskinesia may be misinterpreted as part of the underlying ID and under-recorded, an example of 'diagnostic overshadowing'. Several assessment scales are available for measuring movement side-effects of antipsychotic drugs and may be utilised in monitoring, although there are obvious challenges in assessment of subjective symptoms (such as akathisia) people with ID who may have limited understanding and verbal communication ability.[25,26,27]

Neuroleptic malignant syndrome is a rare idiosyncratic complication of antipsychotic therapy consisting of fever, muscle rigidity, autonomic dysfunction, and alterations in cognitive state. We found a significantly increased incidence of neuroleptic malignant syndrome amongst people with ID, although the low number of recorded events in the database means our results need to be interpreted with caution. An association between neuroleptic malignant syndrome and ID has been demonstrated previously[28,29] and this, combined with the seriousness of the condition (particularly in people with ID[29,30]), warrants further attention.

We observed a decline in the recording of movement side-effects in both groups over the past 15 years. It might be that; clinicians have focused their attention on measuring and managing metabolic complications; the wide scale switch from first- to second-generation antipsychotic drugs[31] has partially contributed to an actual decrease in the rate of movement side-effect; the clinical expectation of reduced movement side-effects with newer drugs has reduced vigilance and recognition of these side-effects.

For peer review only

Strengths and limitations

This is the first study to directly compare the rate of antipsychotic drug-induced movement side-effects between people with and without ID. Our findings are strengthened by the large numbers of people included and the representativeness of the sample drawn from a representative community population. UK general practices are incentivised to maintain an accurate list of people with ID. Prescriptions are also accurately recorded in THIN although by excluding depot preparations (and the small number of prescriptions that may have been issued in secondary care) we might have underestimated exposure to antipsychotic drugs.

A limitation of our work that is common across observational studies that utilise routinely-collected health data is the lack of direct validation of diagnoses. The Read code list for movement side-effects was devised using a comprehensive methodology and with input from practising primary and specialist secondary care physicians but not tested against 'gold standard' methods for identifying movement side-effects. We assume that relevant Read codes added to patient records during exposure to antipsychotic drugs represent adverse side-effects; this may not always be the case and symptoms of movement disorder may arise independently or in response to other prescribed medications (such as anti-depressants and anti-epileptic drugs) that we did not measure. Our method measures only recorded side-effects, that is, people must consult their General Practitioner for the side-effect to be noted formally. Even when seen by a clinician, there is evidence that movement side-effects might be missed.[32] It is possible, therefore, that our results under-estimate the true rate of movement side-effect of antipsychotic drugs. How this might bias the comparison between ID and non-ID groups is not clear. People with ID have lower health literacy, lack knowledge of psychotropic drug side-effects,[33] and may encounter barriers to accessing primary care,[34] and hence be less likely to present to primary care when experiencing treatment side-effects. Conversely, people with ID may be monitored more closely by carers or by pro-active General Practitioners who recognise the higher health need in this group, for example, by offering an annual health check. Some cases of movement side-effects may have been missed if people who are in contact with specialist services contact their psychiatrist directly, rather than visiting their General Practitioner.

For peer review only

Neuroleptic malignant syndrome may be underestimated either because milder forms are missed, or because it is more likely to be treated in the acute hospital.

Further work will be needed to elucidate the potential pathophysiological mechanism underlying the observed association between ID and movement side-effects of antipsychotic drugs.

Conclusions

Movement side-effects are only one aspect of a number of antipsychotic drug adverse effects. They can impact medication compliance, quality of life, and compound the stigma of mental illness and/or intellectual disability.[35,36] They can be difficult to recognise, to treat and, in the case of the tardive syndromes, can persist or even worsen on withdrawal of the offending drug. People with ID appear more susceptible to movement side-effects of antipsychotic drugs than people without ID and this should be considered when treatment decisions are made, especially given the relatively high rates of other comorbidities in this population. Our data support a modest potential benefit of second generation antipsychotic drugs in reducing the incidence of movement side-effects, but more work is needed to confirm this finding, and it must be balanced against the increased propensity of second-generation agents to cause metabolic side-effects.

There has been much recent public and professional interest in the prescription of antipsychotic drugs to people with intellectual disability and UK national policy supports attempts to reduce the prescribing of antipsychotic drugs for challenging behaviour (https://www.england.nhs.uk/wp-content/uploads/2016/06/stopping-over-medication.pdf). We recently showed that reduction or discontinuation of antipsychotic drugs in people with ID and challenging behaviour (but without severe mental illness) risks harm as well as providing potential benefits and advocate individual treatment decisions in this group.[37] The current work informs the risk-benefit analysis undertaken as part of antipsychotic drug prescribing in people with ID and reinforces the need for regular and effective medication review, which must include assessment of possible movement side-effects.

For peer review only

For peer review only

References

1. Marsden C, Jenner P. The pathophysiology of extrapyramidal side-effects of neuroleptic drugs. *Psychological medicine* 1980; **10**: 55-72.

2. Leucht S, Wahlbeck K, Hamann J, Kissling W. New generation antipsychotics versus low-potency conventional antipsychotics: a systematic review and meta-analysis. *The Lancet* 2003; **361**: 1581-9.

3. Cooper S-A, Smiley E, Morrison J, Williamson A, Allan L. Mental ill-health in adults with intellectual disabilities: prevalence and associated factors. *The British Journal of Psychiatry* 2007; **190**: 27-35.

4. Sheehan R, Hassiotis A, Walters K, Osborn D, Strydom A, Horsfall L. Mental illness, challenging behaviour, and psychotropic drug prescribing in people with intellectual disability: UK population based cohort study. *BMJ* 2015; **351**: h4326.

5. Deb S, Sohanpal S, Soni R, Lentre L, Unwin G. The effectiveness of antipsychotic medication in the management of behaviour problems in adults with intellectual disabilities. *Journal of Intellectual Disability Research* 2007; **51**: 766-77.

6. Arnold LE. Clinical pharmacological issues in treating psychiatric disorders of patients with mental retardation. *Annals of clinical psychiatry* 1993; **5**: 189-97.

7. Cunningham-Owens DG. Special populations. A guide to extrapyramidal side-effects of antipsychotic drugs. 2nd ed. Cambridge, UK: Cambridge University Press; 2014.

8. Blak B, Thompson M, Dattani H, Bourke A. Generalisability of The Health Improvement Network (THIN) database: demographics, chronic disease prevalence and mortality rates. *Journal of Innovation in Health Informatics* 2011; **19**: 251-5.

9. Bourke A, Dattani H, Robinson M. Feasibility study and methodology to create a quality-evaluated database of primary care data. *Journal of Innovation in Health Informatics* 2004; **12**: 171-7.

10. Booth N. What are the Read codes? *Health libraries review* 1994; **11**: 177-82.

11. Maguire A, Blak BT, Thompson M. The importance of defining periods of complete mortality reporting for research using automated data from primary care. *Pharmacoepidemiology and drug safety* 2009; **18**: 76-83.

12. Horsfall L, Walters K, Petersen I. Identifying periods of acceptable computer usage in primary care research databases. *Pharmacoepidemiology and drug safety* 2013; **22**: 64-9.

13. Davé S, Petersen I. Creating medical and drug code lists to identify cases in primary care databases. *Pharmacoepidemiology and drug safety* 2009; **18**: 704-7.

For peer review only

14. Lewis JD, Bilker WB, Weinstein RB, Strom BL. The relationship between time since registration and measured incidence rates in the General Practice Research Database. *Pharmacoepidemiology and drug safety* 2005; **14**: 443-51.

15. Townsend P, Phillimore P, Beattie A. Health and deprivation: inequality and the North. Routledge; 1988.

16. Marston L, Nazareth I, Petersen I, Walters K, Osborn DP. Prescribing of antipsychotics in UK primary care: a cohort study. *BMJ Open* 2014; **4**: e006135.

17. Frighi V, Stephenson MT, Morovat A, et al. Safety of antipsychotics in people with intellectual disability. *The British Journal of Psychiatry* 2011; **199**: 289-95.

18. Gagiano C, Read S, Thorpe L, Eerdekens M, Van Hove I. Short-and long-term efficacy and safety of risperidone in adults with disruptive behavior disorders. *Psychopharmacology* 2005; **179**: 629-36.

19. Leucht S, Cipriani A, Spineli L, et al. Comparative efficacy and tolerability of 15 antipsychotic drugs in schizophrenia: a multiple-treatments meta-analysis. *The Lancet* 2013; **382**: 951-62.

20. Peluso MJ, Lewis SW, Barnes TR, Jones PB. Extrapyramidal motor side-effects of first-and second-generation antipsychotic drugs. *The British Journal of Psychiatry* 2012; **200**: 387-92.

21. Scheifes A, Walraven S, Stolker JJ, et al. Movement Disorders in Adults With Intellectual Disability and Behavioral Problems Associated With Use of Antipsychotics. *Journal of clinical psychopharmacology* 2016; **36**: 308-13.

22. de Kuijper G, Mulder H, Evenhuis H, Scholte F, Visser F, Hoekstra PJ. Determinants of physical health parameters in individuals with intellectual disability who use long-term antipsychotics. *Research in developmental disabilities* 2013; **34**: 2799-809.

23. Matson JL, Fodstad JC, Neal D, Dempsey T, Rivet TT. Risk factors for tardive dyskinesia in adults with intellectual disability, comorbid psychopathology, and long-term psychotropic use. *Research in developmental disabilities* 2010; **31**: 108-16.

24. Rogers D, Karki C, Bartlett C, Pocock P. The motor disorders of mental handicap. An overlap with the motor disorders of severe psychiatric illness. *The British Journal of Psychiatry* 1991; **158**: 97-102.

25. Guy W. Abnormal involuntary movement scale. ECDEU Assessment Manual for Psychopharmacology. Washington, DC: US Department of Health, Education and Welfare; 1976.

26. Barnes TR. A rating scale for drug-induced akathisia. *The British Journal of Psychiatry* 1989; **154**: 672-6.

27. Simpson G, Angus J. A rating scale for extrapyramidal side effects. *Acta Psychiatrica Scandinavica* 1970; **45**: 11-9.

For peer review only

28. Viejo L, Morales V, Punal P, Perez J, Sancho R. Risk factors in neuroleptic malignant syndrome. A case–control study. *Acta Psychiatrica Scandinavica* 2003; **107**: 45-9.

29. Shalev A, Munitz H. The neuroleptic malignant syndrome: agent and host interaction. *Acta Psychiatrica Scandinavica* 1986; **73**: 337-47.

30. Boyd RD. Neuroleptic malignant syndrome and mental retardation: review and analysis of 29 cases. *American journal on mental retardation* 1993; **98**: 143-55.

31. Verdoux H, Tournier M, Begaud B. Antipsychotic prescribing trends: a review of pharmaco-epidemiological studies. *Acta Psychiatrica Scandinavica* 2010; **121**: 4-10.

32. Haddad PM, Dursun SM. Neurological complications of psychiatric drugs: clinical features and management. *Human Psychopharmacology: Clinical and Experimental* 2008; **23**: S15-S26.

33. Crossley R, Withers P. Antipsychotic medication and people with intellectual disabilities: their knowledge and experiences. *Journal of Applied Research in Intellectual Disabilities* 2009; **22**: 77-86.

34. Lennox N, Diggens J, Ugoni A. The general practice care of people with intellectual disability: barriers and solutions. *Journal of Intellectual Disability Research* 1997; **41**: 380-90.

35. Schouten HJ, Knol W, Egberts TC, Schobben AF, Jansen PA, van Marum RJ. Quality of life of elderly patients with antipsychotic-induced parkinsonism: A cross-sectional study. *Journal of the American Medical Directors Association* 2012; **13**: 82. e1-. e5.

36. Scheifes A, Walraven S, Stolker JJ, Nijman HL, Egberts TC, Heerdink ER. Adverse events and the relation with quality of life in adults with intellectual disability and challenging behaviour using psychotropic drugs. *Research in developmental disabilities* 2016; **49**: 13-21.

37. Sheehan R, Hassiotis A. Reduction or discontinuation of antipsychotics for challenging behaviour in adults with intellectual disability: a systematic review. *The Lancet Psychiatry* 2016; **4**: 238-56.

For peer review only

TABLE 1 – Cohort characteristics

	ID cohort	Non-ID cohort
Total number (%)	9,039 (21)	34,242 (79)
Male, *n* (%)	5,279 (58)	18,825 (55)
Average age, *yrs* (SD)	42 (16)	44 (16)
Townsend score, *n* (%)		
1	1,403 (16)	4,860 (14)
2	1,752 (19)	5,119 (15)
3	1,948 (22)	6,481 (19)
4	1,942 (22)	8,189 (24)
5	1,563 (17)	7,911 (23)
Missing	417 (5)	1,619 (5)
History of antipsychotic use at cohort entry, *n* (%)	6,684 (74)	16,227 (47)
History of movement disorder at cohort entry, *n* (%)	2,192 (24)	4,946 (14)
History of movement disorder without antipsychotic use at cohort entry, *n* (%)	136/2,355 (6)	1,038/18,015 (6)
History of movement disorder and antipsychotic use at cohort entry, *n* (%)	2,056/6,684 (31)	3,908/16,227 (24)
Total person-years between first and last antipsychotic prescription	44,696	104,014
Median years between first and last prescription (IQR)	3.5 (1.2 to 7.9)	1.3 (0.19 to 4.4)
Median years on treatment between first and last prescription (IQR)	2.6 (0.76 to 6.3)	0.67 (0.15 to 2.5)
Average daily dose, *CLZE* (SD)	135 (156)	139 (146)

CLZE, Chlorpromazine equivalents

For peer review only

TABLE 2 – frequency of antipsychotic drugs prescribed in the intellectually disabled and non-intellectually disabled cohorts

Antipsychotic drug	ID cohort				Non-ID cohort			
	Number	Percentage	Average daily dose, mg	Median treatment duration, yrs	Number	Percentage	Average daily dose, mg	Median treatment duration, yrs
Risperidone	4,013	28.5	1.9	1.81	7,426	14.7	2.2	0.47
Olanzapine	2,086	14.8	8.2	1.62	10,246	20.3	8.5	0.67
Chlorpromazine	1,770	12.6	78.5	1.12	5,274	10.5	64.8	0.20
Quetiapine	1,295	9.2	154.1	0.93	7,693	15.2	152.6	0.72
Haloperidol	1,231	8.7	4.9	0.68	3,755	7.5	3.2	0.14
Thioridazine	838	6.0	77.9	0.82	2,672	5.3	49.8	0.20
Aripiprazole	661	4.7	10.5	0.74	2,638	5.2	11.8	0.54
Trifluoperazine	456	3.2	6.5	1.06	3,403	6.8	4.5	0.19
Zuclopenthixol	429	3.1	18.7	2.17	340	0.7	20.0	0.45
Amisulpride	327	2.3	295.0	0.94	1,687	3.4	290.3	0.73
Promazine	276	2.0	58.4	0.33	1,688	3.4	58.6	0.16
Sulpiride	276	2.0	437.8	1.97	1,199	2.4	435.6	0.79
Other*	980	7.0	-	-	5,230	10.4	-	-

*Other antipsychotic drugs prescribed to <1% of ID cohort each

For peer review only

TABLE 3 – Incidence rates and adjusted incident rate ratios for movement side-effects in people with and without intellectual disability prescribed antipsychotic drugs

	**ID cohort**			**Non-ID cohort**			**Comparison**	
**Variable**	No of events during follow-up	No of Person-Years (X10,000)	Incidence per 10,000 Person-Years (95%CI)	No of events during follow-up	No of Person-Years (X10,000)	Incidence per 10,000 Person-Years (95%CI)	Incidence Rate Ratio* (95%CI)	p-value
**Any movement disorder (defined by Read code or antimuscarinic prescription)**	**743**	**2.7**	**275 (256 to 296)**	**1750**	**7.0**	**248 (237 to 260)**	**1.30 (1.18 to 1.42)**	**<0.001**
**Any movement disorder (defined by Read code)**	**446**	**4.4**	**111 (101 to 122)**	**952**	**9.4**	**101 (95 to 108)**	**1.30 (1.16 to 1.47)**	**<0.001**
**Any movement disorder (defined by antimuscarinic prescription)**	**564**	**2.9**	**196 (180 to 212)**	**1299**	**7.6**	**172 (163 to 181)**	**1.29 (1.16 to 1.44)**	**<0.001**
Acute dystonia	60	4.4	14 (11 to 18)	161	10.2	16 (14 to 19)	1.00 (0.73 to 1.37)	0.99
**Akathisia**	**80**	**4.5**	**18 (15 to 23)**	**112**	**10.3**	**11 (9 to 13)**	**2.29 (1.69 to 3.12)**	**<0.001**
**Pseudo-Parkinsonism**	**270**	**4.4**	**64 (57 to 72)**	**592**	**9.9**	**60 (55 to 65)**	**1.20 (1.03 to 1.39)**	**0.02**
Tardive dyskinesia	61	4.0	14 (11 to 18)	123	10.3	12 (10 to 14)	1.27 (0.91 to 1.75)	0.16
**Neuroleptic malignant syndrome**	**11**	**4.4**	**3 (1 to 5)**	**12**	**10.4**	**1 (1-2)**	**3.03 (1.26 to 7.30)**	**0.013**
Other movement disorder	43	4.2	10 (7 to 13)	94	10.3	9 (7-11)	1.26 (0.86 to 1.85)	0.23

*Adjusted for sex, social deprivation score, time period, history of antipsychotic drug use, average daily dose, days on treatment

For peer review only

TABLE 4 – Sensitivity analysis with incidence rates and adjusted incident rate ratios for movement side-effects in people with and without intellectual disability prescribed first- and second-generation antipsychotic drugs

	**ID cohort**			**Non-ID cohort**			**Comparison**	
**Antipsychotic class**	No of events during follow-up	No of Person-Years (X10,000)	Incidence per 10,000 Person-Years (95%CI)	No of events during follow-up	No of Person-Years (X10,000)	Incidence per 10,000 Person-Years (95%CI)	Incidence Rate Ratio* (95%CI)	p-value
**First generation****	**247**	**0.8**	**320 (283 to 362)**	**569**	**1.9**	**293 (270 to 318)**	**1.36 (1.16 to 1.60)**	**<0.001**
**Second generation****	**378**	**1.6**	**241 (218 to 267)**	**948**	**4.3**	**219 (206 to 233)**	**1.43 (1.26 to 1.62)**	**<0.001**
**Risperidone****	**124**	**0.6**	**196 (164 to 233)**	**96**	**0.5**	**182 (149 to 223)**	**1.55 (1.15 to 2.08)**	**0.004**

*Adjusted for sex, social deprivation score, time period, history of antipsychotic drug use, average daily dose, days on treatment

** Restricted to periods when people were exclusively prescribed first- or second-generation antipsychotic drugs or risperidone

For peer review only

FIGURE 1 – Time trends in crude incidence rates of a) movement side-effect defined by Read code and b) antimuscarinic drug prescription in people with and without intellectual disability prescribed antipsychotic drugs

a)

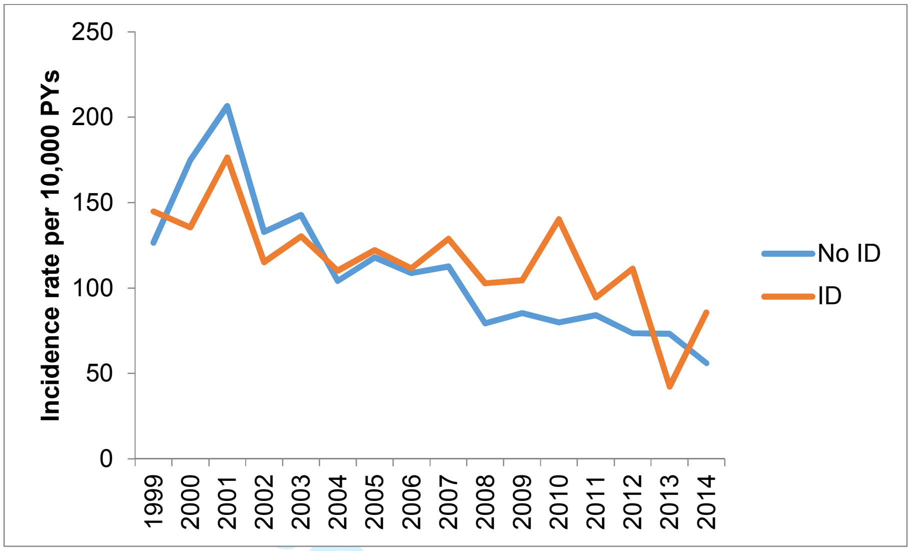

		p-value	95% CI
**Non-ID group**	0.93	<0.0001	0.91 to 0.95
**ID**	0.95	0.00064	0.92 to 0.98

b)

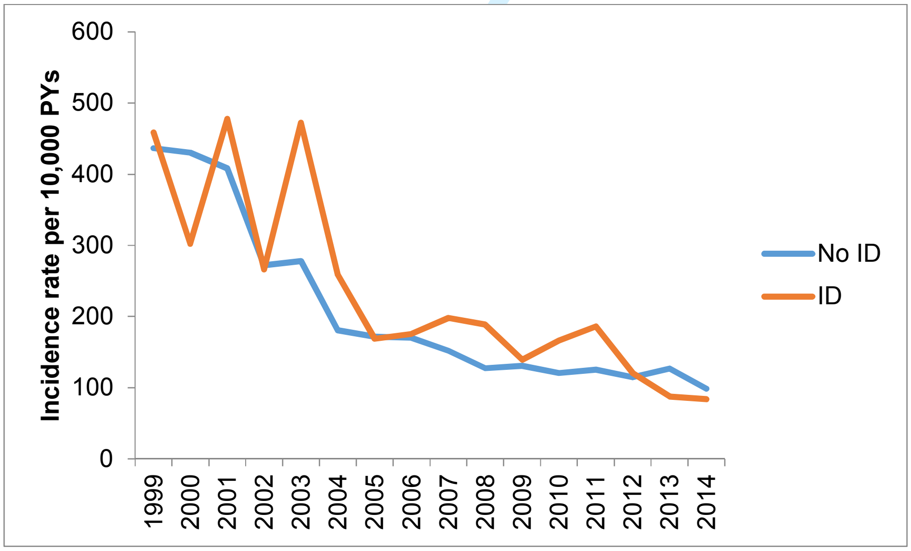

		p-value	95% CI
**Non-ID group**	0.95	<0.0001	0.93 to 0.96
**ID group**	0.97	0.002	0.95 to 0.99

Per year time trends adjusted for sex, social deprivation, history of antipsychotic use, average daily dose in Chlorpromazine equivalents, days on treatment

For peer review only - http://bmjopen.bmj.com/site/about/guidelines.xhtml

STROBE Statement—checklist of items that should be included in reports of observational studies

Movement side-effects of antipsychotic drugs in adults with intellectual disability

	Item No.	Recommendation	Page No.	Relevant text from manuscript
**Title and abstract**	1	(*a*) Indicate the study's design with a commonly used term in the title or the abstract	1	Cohort study in title
		(*b*) Provide in the abstract an informative and balanced summary of what was done and what was found	2	
**Introduction**				
Background/rationale	2	Explain the scientific background and rationale for the investigation being reported	6	"People with ID are often considered to be at greater risk of antipsychotic drug-induced movement side-effects than people without ID but no studies directly compare rates"
Objectives	3	State specific objectives, including any prespecified hypotheses	7	"To compare the incidence of recorded movement side-effects in adults with and without ID who were prescribed antipsychotic drugs".
**Methods**				
Study design	4	Present key elements of study design early in the paper	7-9	Data source, study cohort, covariates subheadings
Setting	5	Describe the setting, locations, and relevant dates, including periods of recruitment, exposure, follow-up, and data collection	7-8	Study cohort subheading
Participants	6	(*a*) *Cohort study*—Give the eligibility criteria, and the sources and methods of selection of participants. Describe methods of follow-up<br>*Case-control study*—Give the eligibility criteria, and the sources and methods of case ascertainment and control selection. Give the rationale for the choice of cases and controls<br>*Cross-sectional study*—Give the eligibility criteria, and the sources and methods of selection of	7-8	Study cohort subheading "all adults with ID…were extracted"

For peer review only - http://bmjopen.bmj.com/site/about/guidelines.xhtml

		participants		
		(*b*) *Cohort study*—For matched studies, give matching criteria and number of exposed and unexposed<br>*Case-control study*—For matched studies, give matching criteria and the number of controls per case	8	"A comparison cohort…was extracted using stratified sampling within each GP practice with frequency matching…"
Variables	7	Clearly define all outcomes, exposures, predictors, potential confounders, and effect modifiers. Give diagnostic criteria, if applicable	7-9	Read code lists described<br>Covariates described
Data sources/ measurement	8*	For each variable of interest, give sources of data and details of methods of assessment (measurement). Describe comparability of assessment methods if there is more than one group	7	Data source subheading
Bias	9	Describe any efforts to address potential sources of bias	8	"Comparison cohort matched to ensure similar population level characteristics across the two groups"
Study size	10	Explain how the study size was arrived at	7	"All adults with recorded ID"

Continued on next page

For peer review only - http://bmjopen.bmj.com/site/about/guidelines.xhtml

Quantitative variables	11	Explain how quantitative variables were handled in the analyses. If applicable, describe which groupings were chosen and why	9	Statistical analysis subheading
Statistical methods	12	(*a*) Describe all statistical methods, including those used to control for confounding	9	"Multivariable mixed Poisson regression was used…"
		(*b*) Describe any methods used to examine subgroups and interactions		
		(*c*) Explain how missing data were addressed	9	"It was not possible to estimate the daily dose for 5% of prescriptions and these were excluded from the study".
		(*d*) *Cohort study*—If applicable, explain how loss to follow-up was addressed<br>*Case-control study*—If applicable, explain how matching of cases and controls was addressed<br>*Cross-sectional study*—If applicable, describe analytical methods taking account of sampling strategy	NA	
		(*e*) Describe any sensitivity analyses	9	"We conducted a sensitivity analysis…"
**Results**				
Participants	13*	(a) Report numbers of individuals at each stage of study—eg numbers potentially eligible, examined for eligibility, confirmed eligible, included in the study, completing follow-up, and analysed	NA	
		(b) Give reasons for non-participation at each stage	NA	
		(c) Consider use of a flow diagram	NA	
Descriptive data	14*	(a) Give characteristics of study participants (eg demographic, clinical, social) and information on exposures and potential confounders	10<br>Table 1	
		(b) Indicate number of participants with missing data for each variable of interest		
		(c) *Cohort study*—Summarise follow-up time (eg, average and total amount)	Table 1	Total person years between first and last prescription, median years between first and last prescription
Outcome data	15*	*Cohort study*—Report numbers of outcome events or summary measures over time	10<br>Table 3	"The overall incidence of recorded movement side effects…"
		Case-control study—Report numbers in each exposure category, or summary measures of exposure	NA	
		Cross-sectional study—Report numbers of outcome events or summary measures	NA	
Main results	16	(*a*) Give unadjusted estimates and, if applicable, confounder-adjusted estimates and their precision	10	

For peer review only - http://bmjopen.bmj.com/site/about/guidelines.xhtml

	(eg, 95% confidence interval). Make clear which confounders were adjusted for and why they were included	Table 2
	(*b*) Report category boundaries when continuous variables were categorized	NA
	(*c*) If relevant, consider translating estimates of relative risk into absolute risk for a meaningful time period	

Continued on next page

For peer review only

Other analyses	17	Report other analyses done—eg analyses of subgroups and interactions, and sensitivity analyses	11<br>Table 4	"Analysis restricted to periods when people were exclusively prescribed first- or second-generation antipsychotic drugs did not change the results".
**Discussion**				
Key results	18	Summarise key results with reference to study objectives	11	"Our data suggest that people with ID are indeed more likely to experience movement side-effects of antipsychotic drugs".
Limitations	19	Discuss limitations of the study, taking into account sources of potential bias or imprecision. Discuss both direction and magnitude of any potential bias	14-15	
Interpretation	20	Give a cautious overall interpretation of results considering objectives, limitations, multiplicity of analyses, results from similar studies, and other relevant evidence	15	"People with ID appear more susceptible to movement side-effects of antipsychotic drugs than people without ID".
Generalisability	21	Discuss the generalisability (external validity) of the study results		
**Other information**				
Funding	22	Give the source of funding and the role of the funders for the present study and, if applicable, for the original study on which the present article is based	5	"The Baily Thomas Charitable Fund and the National Institute for Health Research"

*Give information separately for cases and controls in case-control studies and, if applicable, for exposed and unexposed groups in cohort and cross-sectional studies.

Note: An Explanation and Elaboration article discusses each checklist item and gives methodological background and published examples of transparent reporting. The STROBE checklist is best used in conjunction with this article (freely available on the Web sites of PLoS Medicine at http://www.plosmedicine.org/, Annals of Internal Medicine at http://www.annals.org/, and Epidemiology at http://www.epidem.com/). Information on the STROBE Initiative is available at www.strobe-statement.org.

For peer review only - http://bmjopen.bmj.com/site/about/guidelines.xhtml

Movement side-effects of antipsychotic drugs in adults with and without intellectual disability: UK population-based cohort study

Journal:	*BMJ Open*
Manuscript ID	bmjopen-2017-017406.R1
Article Type:	Research
Date Submitted by the Author:	02-Jun-2017

Complete List of Authors:	Sheehan, Rory; University College London, Division of Psychiatry<br>Horsfall, Laura; University College London, Research Department of Primary Care and Population Health<br>Strydom, Andre; Division of Psychiatry<br>Osborn, David; University College London , Division of Psychiatry<br>Walters, Kate; University College London, Research Department of Primary Care and Population Health<br>HASSIOTIS, ANGELA; University College London, Division of Psychiatry

**Primary Subject Heading**:	Mental health
Secondary Subject Heading:	Pharmacology and therapeutics
Keywords:	MENTAL HEALTH, PSYCHIATRY, Adverse events < THERAPEUTICS

SCHOLARONE™ Manuscripts

For peer review only

TITLE PAGE

Movement side-effects of antipsychotic drugs in adults with and without intellectual disability: UK population-based cohort study

Dr Rory Sheehan*, MSc, Division of Psychiatry, University College London

Dr Laura Horsfall*, PhD, Research Department of Primary Care and Population Health, University College London

Dr André Strydom, PhD, Division of Psychiatry, University College London

Professor David Osborn, PhD, Division of Psychiatry, University College London

Dr Kate Walters, PhD, Research Department of Primary Care and Population Health, University College London

Professor Angela Hassiotis, PhD, Division of Psychiatry, University College London

*Rory Sheehan and Laura Horsfall contributed equally to this work.

Corresponding author: Dr Rory Sheehan

Address: Division of Psychiatry, University College London, 6th Floor Maple House, 149 Tottenham Court Road, London W1T 7NF

Telephone: 0207-679-9201

E-mail: r.sheehan@ucl.ac.uk

For peer review only

ABSTRACT

Movement side-effects of antipsychotic drugs in adults with and without intellectual disability: UK population-based cohort study

Objectives To measure the incidence of movement side-effects of antipsychotic drugs in adults with intellectual disability and compare rates with adults without intellectual disability.

Design Cohort study using data from The Health Improvement Network

Setting UK Primary care

Participants Adults with intellectual disability prescribed antipsychotic drugs matched to a control group of adults without intellectual disability prescribed antipsychotic drugs.

Outcome measures New records of movement side-effect including, acute dystonias, akathisia, Parkinsonism, tardive dyskinesia, and neuroleptic malignant syndrome.

Results 9,013 adults with intellectual disability and a control cohort of 34,242 adults without intellectual disability together contributed 148,709 person-years data. The overall incidence of recorded movement side-effects was 275 per 10,000 person-years (95% confidence interval 256-296) in the intellectual disability group and 248 per 10,000 person-years (95% confidence interval 237-260) in the control group. The incidence of any recorded movement side-effect was significantly greater in people with intellectual disability compared to those without (incidence rate ratio 1.30, 95% confidence interval 1.18 to 1.42, p<0.001, after adjustment for potential confounders), with Parkinsonism and akathisia showing the greatest difference between the groups. Neuroleptic malignant syndrome, although occurring infrequently, was three times more common in people with intellectual disability prescribed antipsychotic drugs (incidence rate ratio 3.03, 95% confidence interval 1.26 to 7.30, p=0.013). Differences in rates of movement side-effects between the groups were not

For peer review only

due to differences in the proportions prescribed first- and second-generation antipsychotic drugs.

Conclusions This study provides evidence to substantiate the long-held assumption that people with intellectual disability are more susceptible to movement side-effects of antipsychotic drugs. Assessment for movement side-effects should be integral to antipsychotic drug monitoring in people with intellectual disability. Regular medication review is essential to ensure optimal prescribing in this group.

For peer review only

ARTICLE SUMMARY

Strengths and limitations of this study

- This study includes a very large number of people with and without intellectual disability who have been prescribed antipsychotic drugs.
- The Health Improvement Network is a UK primary care database that contains accurate recording of demographic and clinical information and drug prescribing.
- This is the first study to directly compare the rates of movement side-effects of antipsychotic drugs between people with intellectual disability and those without, and offers new insights into the risk-benefit ratio of antipsychotic drug prescribing to people with intellectual disability.
- Recording of movement side-effects of antipsychotic drugs in primary care has not been validated.
- Antipsychotic drugs prescribed outside primary care and movement side-effects identified in other settings may not have been recorded by our method.

For peer review only

FUNDING STATEMENT

This work was supported by The Baily Thomas Charitable Fund grant number 518669 and the National Institute for Health Research grant reference RP-DG-0611-10003. The funders had no role in study design, data collection and analysis, decision to publish, or preparation of the manuscript.

COMPETING INTERESTS

Rory Sheehan reports no conflicts of interest.

Laura Horsfall reports no conflicts of interest.

André Strydom reports grants from Roche pharmaceuticals, outside the submitted work.

David Osborn reports no conflicts of interest.

Kate Walters reports no conflicts of interest.

Angela Hassiotis reports no conflicts of interest.

AUTHOR CONTRIBUTIONS

Rory Sheehan developed the idea and method for the study, interpreted the results, and wrote the manuscript. Rory Sheehan is guarantor.

Laura Horsfall developed the idea and method for the study, performed the data extraction and analysis, interpreted the results, and wrote the manuscript.

André Strydom developed the idea and method for the study, interpreted the results, and wrote the manuscript.

David Osborn developed the idea and method for the study, interpreted the results, and wrote the manuscript.

Kate Walters developed the idea and method for the study, interpreted the results, and wrote the manuscript.

Angela Hassiotis developed the idea and method for the study, interpreted the results, and wrote the manuscript.

DATA SHARING STATEMENT

Copies of Read code lists used in this study are available from the authors, on request.

For peer review only

MANUSCRIPT

Movement side-effects of antipsychotic drugs in adults with and without intellectual disability: UK population-based cohort study

Introduction

Movement (extra-pyramidal) side-effects, including acute dystonias, akathisia, Parkinsonism, and tardive dyskinesia, are a well-recognised complication of antipsychotic drugs which are thought to occur secondary to antagonism of dopamine D2 receptors in the striatum and meso-cortex.[1] Movement side-effects can be distressing, disabling, and difficult to treat and their presence is associated with poor medication compliance, stigma, and reduced quality of life.[2]

Intellectual disability (ID) is a lifelong condition characterised by global deficits in cognitive and adaptive functioning. People with ID experience relatively high rates of mental illness[3] and many worldwide and in the UK are prescribed antipsychotic drugs. There has been renewed focus on the appropriateness of antipsychotic drug prescribing in people with ID following recent evidence that antipsychotic drugs are often used in the absence of an underlying diagnosed mental illness,[4] in many cases in an attempt to manage challenging behaviour, despite a lack of evidence that they are effective in this context.[5] There has been relatively little formal investigation of antipsychotic drug side-effects in people with ID and most of our knowledge is extrapolated from studies conducted in people of average intelligence. People with ID are often considered to be at greater risk of antipsychotic drug-induced movement side-effects than people without ID[6] but no studies directly compare rates between the two groups. Furthermore, knowledge of a specific mechanism that might underpin any association between ID and movement side-effects extends only to a vague theory that organic brain dysfunction makes centrally-mediated side-effects of psychotropic drugs more likely.[7]

For peer review only

We undertook a cohort study using a large nationally-representative database to compare the incidence of recorded movement side-effects in adults with and without ID who are prescribed antipsychotic drugs.

Methods

Data source

Data were obtained from The Health Improvement Network (THIN), a large UK primary care database that contains the electronic health records of more than 3.7 million active patients in over 550 general practices (http://www.epic-uk.org/our-data/our-data.shtml). The patients included in the database are representative of the UK population in age, sex, and morbidity and mortality.[8,9] The vast majority of people with intellectual disability in the UK live in the community and are registered with a General Practitioner (primary care physician) who provides routine and ongoing care, and who acts as gatekeeper for hospital-based specialists, including psychiatrists. The THIN database contains clinical records added by General Practitioners using a clinical dictionary of Read codes. Read codes are standardised clinical terms that can be used as shorthand for clinicians to record certain patient characteristics (such as occupation and living circumstances) and the content of a consultation.[10] Individual Read codes exist to cover the variety of signs, symptoms and diagnoses that an individual may have, as well as test results and surgical or therapeutic treatments. Recording of illness in primary care records has been shown to be accurate and all prescribed medication must be issued through the electronic system. National Health Service drug budgets flow through primary care and General Practitioners issue most prescriptions directly, including those for psychotropic drugs. The primary care record therefore is a suitable means of conducting pharmaco-epidemiological research.

THIN data are pseudonymised at source and made available to researchers who have purchased a license. THIN has overall ethical approval to collate data and this study received approval from the THIN Scientific Review Committee (reference 14-071).

Study cohort

For peer review only - http://bmjopen.bmj.com/site/about/guidelines.xhtml

For this study, all adults with recorded ID and a history of oral antipsychotic drug prescription were extracted using a previously-defined and tested list of diagnostic Read codes (including codes for ID and conditions associated with ID) and antipsychotic drug codes,[4] based on chapters of the *British National Formulary*. General practitioners are incentivised by the Quality Outcomes Framework (QOF) to keep a register of people with ID which improves recording in the database. The study period was 1st January 1999 to 31st December 2014. Entry to the cohort was set as the date of the first antipsychotic drug prescription issued after the latest of; registration with the GP practice contributing data; the patient's 18th birthday; the start of the study period; the date the practice achieved compliance with standard measures of data quality.[11,12] People contributed person-years (PYs) of data from entry to cohort exit. Exit from the cohort was defined as the first of; the final antipsychotic drug prescription plus the length of the prescription; de-registration with the GP practice contributing data; the end of the study period; or death.

A comparison cohort of people prescribed antipsychotic drugs but without ID was extracted using stratified sampling within each GP practice with frequency matching to ensure similar population-level characteristics across the two cohorts in terms of age, gender, and year of antipsychotic prescription. Up to six people without ID were selected for every person with ID and the same criteria were used to define cohort entry and exit.

A Read code list for movement side-effects was developed using previously-described methodology and applied to the cohort to determine incidence of movement side-effects[13] (supplementary data). Movement side-effects were categorised as; acute dystonias; akathisia; Parkinsonism; or tardive dyskinesia; in accordance with orthodox classification. A separate category was established for neuroleptic malignant syndrome (NMS), being a very specific adverse effect, and a further category included for broad codes which could not be sub-categorised. Prescriptions for selected antimuscarinic drugs (procyclidine, orphenadrine, trihexyphenidyl) were used as proxy indicators of movement side-effects in those prescribed antipsychotic drugs. People were defined as having a history of movement disorder if a relevant Read code was applied (or antimuscarinic drug prescribed) prior to

For peer review only

cohort entry or within six months of registration with the practice, as this has been shown to improve the validity of incidence calculations.[14]

Covariates

Sociodemographic covariates included age, sex, calendar year, and the Townsend Deprivation Score (a composite score in fifths based on postcode and Census recording of local unemployment, car ownership, home ownership, and overcrowding).[15] Other covariates included history of antipsychotic use at cohort entry, antipsychotic average daily dose, and days on treatment between the start and end of follow-up. Average daily dose was measured as Chlorpromazine equivalents (CPZE) to account for polypharmacy and those who switched drugs during follow-up. Where we were unable to extract daily dose data (for example, in the minority of cases where the duration of a prescription was not recorded) the prescribed dose for the previous or subsequent prescription for the same drug and formulation was used. It was not possible to estimate the daily dose for 5% of prescriptions and these were excluded from the study. Prescriptions for drug doses above three times the upper licensed limit were excluded as probable coding errors (<1%).

Statistical analysis

Multivariable mixed Poisson regression was used to calculate incident rates of movement disorders during exposure to antipsychotic drugs in people with and without ID by calendar year, adjusted for any temporal changes in age and sex. Incidence rate was defined as the number of new events of interest / the duration that the cohort was at risk. First we were interested in the incidence of new cases of any movement disorder. Participants exited the cohort when they were first diagnosed with any movement disorder as they were no longer considered at risk of a new diagnosis after this date. For calculating the incidence of subtypes of movement side-effect, participants exited the cohort after they were diagnosed with the type of movement side-effect of interest as they were no longer considered at risk of that type of movement side-effect after that date. They remained in the cohort for the purposes of being diagnosed with other types of movement side-effect as an individual participant may develop more than one type of movement side-effect of antipsychotic drug.

For peer review only

General practice was included as a random effect to account for any data clustering. Calendar year was initially modelled as a continuous variable and we then used the likelihood ratio test to compare this with a model in which year was entered as a categorical variable to examine the possibility of non-linear time trends.

Multivariable mixed Poisson regression was also used to compare the rates of movement disorder during exposure to antipsychotic drugs in people with and without ID, adjusted for covariates. We conducted a sensitivity analysis where we restricted the analysis to time periods when people were exclusively prescribed first- or second-generation antipsychotic drugs, and further when we restricted the analysis to times when only risperidone was prescribed. All analyses were repeated after excluding people with a diagnosis of idiopathic Parkinson's disease.

We considered a p-value of 0.05 to be statistically significant (two-tailed) and used Stata version 13 for all analyses (StataCorp, TX).

Results

In total 9,039 people with ID met inclusion criteria and were matched to 34,242 people without ID, and together contributed 148,709 person-years (PYs) data. The two cohorts were similar in terms of age, sex, level of social deprivation, and history of movement disorder at cohort entry (table 1). The prevalence of movement disorder at baseline was slightly higher for people with ID and a history of antipsychotic use (31%) compared to those without ID and a history of antipsychotic drug use (24%) but the proportions of those with a history of movement disorder without antipsychotic drug use were equal (6%) at cohort entry. Average daily dose of antipsychotic was similar between the two groups but those with ID had longer time periods between their first and last antipsychotic prescription and more days on treatment between those dates. Table 2 shows the distribution of antipsychotic drugs prescribed to the study cohort. Risperidone was the most common drug prescribed in the ID cohort (28.5% prescriptions to people with ID, 14.7% prescriptions in

For peer review only

the non-ID cohort); other drugs were prescribed in roughly equal proportions between the two groups.

[Table 1 near here]

[Table 2 near here]

The overall incidence of recorded movement side-effects was 275 per 10,000PYs (95% confidence interval (CI) 256-296) in the ID group and 248 per 10,000PYs (95%CI 237-260) in the non-ID group (table 3). Parkinsonism was the most commonly recorded movement side-effect in both groups. After adjustment, the incidence rate of any movement disorder was 30% higher in people with ID compared to those without ID (IRR 1.30, 95%CI 1.18 to 1.42, p<0.001). Similar differences in movement side-effect recording were noted when defined by diagnostic Read codes or by proxy, using prescription for antimuscarinic drugs. The incidence rates of akathisia, Parkinsonism, and neuroleptic malignant syndrome were significantly higher in those with ID compared with those without ID.

Analysis restricted to periods when people were exclusively prescribed first- or second-generation antipsychotic drugs did not change the results; movement side-effects were still significantly more likely to be recorded in people with ID compared to those without (table 4). Excluding people with Parkinson's disease from the analysis (n=451) had no meaningful effect on any of the results.

[Table 3 near here]

[Table 4 near here]

Time trend analysis showed that the incidence of recording of movement side-effects in those prescribed antipsychotic drugs fell significantly over the course of the study period in both groups (figure 1); each calendar year was associated with a 5% decline in the recording of movement side-effects in people with ID (95%CI 2-8%, p<0.0001) and a 7% decline in people without ID (95%CI 5-9%, p<0.001), after accounting for changes in cohort age and

For peer review only

sex. Prescriptions for antimuscarinic drugs declined by 3% per year in people with ID (95%CI 1-5%, p=0.002) and 5% per year in people without ID (95%CI 4-7%, p<0.001). Average daily antipsychotic dose, measured in Chlorpromazine equivalents, remained broadly constant during the study period (data not shown).

[Figure 1a and figure 1b near here]

Discussion

People with ID have long been considered at greater risk of adverse side-effects of antipsychotic drugs. However to date very little evidence has been presented to substantiate this belief. Our data suggest that people with ID are more likely to experience movement side-effects of antipsychotic drugs; this finding is robust and persists when movement side-effects are defined by diagnostic Read code alone and when they are measured using prescription of anti-muscarinic drugs as proxy.

Most people in our cohort received a second-generation antipsychotic drug. The types of drugs that were prescribed in our study were broadly similar between the group with ID and the group without, and were consistent with other recent data examining antipsychotic prescribing in community-dwelling adults living in the UK.[16] The exception was risperidone, which was prescribed more frequently to people with ID; this accords with other studies[17] and is likely to reflect an attempt by clinicians to prescribe the antipsychotic with greatest (albeit still limited) evidence of benefit for challenging behaviour in people with ID.[5,18] The difference in incidence of movement side-effects between people with and without ID remained when first- and second-generation agents were considered separately, and when risperidone was considered alone, suggesting that headline differences between the groups were not due to different prescribing practices.

Although their improved movement side-effect profile has been considered a major advantage of second-generation antipsychotic drugs, more recent evaluation of the evidence suggests that the initial enthusiasm for second-generation agents was misplaced and largely based on studies that made unequal comparisons between second-generation

For peer review only

and high-potency first-generation drugs.[19,20] In this study we did not set out to compare movement side-effects between first and second-generation agents but we observed that the prescription of second-generation agents was associated with a slightly lower incidence of recorded movement side-effects in people with and without ID; clearly further work is needed to provide definitive data on this contentious aspect of antipsychotic drug side-effects.

There is a lack of work with which we can directly compare our results; previous studies that investigate movement disorders in people with ID who take anti-psychotic drugs have used differing methods to define and ascertain movement disorder, selected particular populations (often convenience sampling those residing in institutions), and tend to report point prevalence figures. None have directly compared rates of movement side-effect in people with ID to controls without ID. Nevertheless, it is clear that antipsychotic drug-induced movement side-effects are reasonably common in people with ID. In once recent study of hospitalised patients with borderline-mild ID and challenging behaviour, almost half were found to have a movement disorder, and the presence of movement disorder was more likely in those prescribed antipsychotic medication.[21] The most common type was Parkinsonism, as in our study. De Kuijper and colleagues in the Netherlands report that just over half of their sample with ID who had been taking antipsychotic drugs for more than a year had evidence of movement side-effects.[22]

It is interesting that in our study the rates of recording of tardive dyskinesia were relatively low and it was not more frequently recorded in people with ID. Previous work has shown tardive dyskinesia to be common; one study of institutionalised adults with ID taking long-term antipsychotic drugs found a prevalence rate of tardive dyskinesia of 45%.[23] Spontaneous dyskinesias are also common in people with ID[24] and it is possible that drug-induced tardive dyskinesia may be misinterpreted as part of the underlying ID and under-recorded, an example of ‘diagnostic overshadowing’. Conversely, it is also possible that background dyskinesia related to ID might be misinterpreted as being the result of antipsychotic drugs. Several assessment scales are available for measuring movement side-effects of antipsychotic drugs and may be utilised in monitoring, although there are obvious

For peer review only

challenges in assessment of subjective symptoms (such as akathisia) people with ID who may have limited understanding and verbal communication ability.[25,26,27]

Neuroleptic malignant syndrome is a rare idiosyncratic complication of antipsychotic therapy consisting of fever, muscle rigidity, autonomic dysfunction, and alterations in cognitive state. We found a significantly increased incidence of neuroleptic malignant syndrome amongst people with ID, although the low number of recorded events in the database means our results need to be interpreted with caution. An association between neuroleptic malignant syndrome and ID has been demonstrated previously[28,29] and this, combined with the seriousness of the condition (particularly in people with ID[29,30]), warrants further attention.

We observed a decline in the recording of movement side-effects in both groups over the past 15 years. It might be that; clinicians have focused their attention on measuring and managing metabolic complications; the wide scale switch from first- to second-generation antipsychotic drugs[31] has partially contributed to an actual decrease in the rate of movement side-effect; the clinical expectation of reduced movement side-effects with newer drugs has reduced vigilance and recognition of these side-effects.

Strengths and limitations

This is the first study to directly compare the rate of antipsychotic drug-induced movement side-effects between people with and without ID. Our findings are strengthened by the large numbers of people included and the sample being drawn from a representative community population. UK general practices are incentivised to maintain an accurate list of people with ID and as prescriptions are also accurately recorded in THIN our results are generalisable across settings to all people with intellectual disability who take antipsychotic drugs. We excluded depot antipsychotic preparations (and the small number of prescriptions that may have been issued in secondary care) and therefore might have slightly underestimated exposure to antipsychotic drugs.

For peer review only

A limitation of our work that is common across observational studies that utilise routinely-collected health data is the lack of direct validation of diagnoses. The Read code list for movement side-effects was devised using a comprehensive methodology and with input from practising primary and specialist secondary care physicians but not tested against 'gold standard' methods for identifying movement side-effects. We assume that relevant Read codes added to patient records during exposure to antipsychotic drugs represent adverse side-effects; this may not always be the case and symptoms of movement disorder may arise independently or in response to other prescribed medications (such as anti-depressants and anti-epileptic drugs) that we did not measure. Our method measures only recorded side-effects, that is, people must consult their General Practitioner for the side-effect to be noted formally. Even when seen by a clinician, there is evidence that movement side-effects might be missed.[32] It is possible, therefore, that our results under-estimate the true rate of movement side-effect of antipsychotic drugs. How this might bias the comparison between ID and non-ID groups is not clear. People with ID have lower health literacy, lack knowledge of psychotropic drug side-effects,[33] and may encounter barriers to accessing primary care,[34] and hence be less likely to present to primary care when experiencing treatment side-effects. Conversely, people with ID may be monitored more closely by carers or by pro-active General Practitioners who recognise the higher health need in this group, for example, by offering an annual health check. Some cases of movement side-effects may have not been recorded in the primary care database if people who are in contact with specialist services contact their psychiatrist directly, rather than visiting their General Practitioner. Neuroleptic malignant syndrome may be underestimated either because milder forms are missed, or because it is more likely to be treated in the acute hospital.

Further work will be needed to elucidate the potential pathophysiological mechanism underlying the observed association between ID and movement side-effects of antipsychotic drugs.

Conclusions

For peer review only

Movement side-effects are only one aspect of a number of antipsychotic drug adverse effects. They can impact medication compliance, quality of life, and compound the stigma of mental illness and/or intellectual disability.[35,36] They can be difficult to recognise, to treat and, in the case of the tardive syndromes, can persist or even worsen on withdrawal of the offending drug. People with ID appear more susceptible to movement side-effects of antipsychotic drugs than people without ID and this should be considered when treatment decisions are made, especially given the relatively high rates of other comorbidities in this population. There is evidence that movement side-effects of antipsychotic drugs are poorly assessed in people with ID who are under the care of secondary care services[37]; this situation must change if medication is to be used in the safest and most effective way possible.

Our data support a modest potential benefit of second generation antipsychotic drugs in reducing the incidence of movement side-effects, but more work is needed to confirm this finding, and it must be balanced against the increased propensity of second-generation agents to cause metabolic side-effects.

There has been much recent public and professional interest in the prescription of antipsychotic drugs to people with intellectual disability and UK national policy supports attempts to reduce the prescribing of antipsychotic drugs for challenging behaviour (https://www.england.nhs.uk/wp-content/uploads/2016/06/stopping-over-medication.pdf). We recently showed that reduction or discontinuation of antipsychotic drugs in people with ID and challenging behaviour (but without severe mental illness) risks harm as well as providing potential benefits and advocate individual treatment decisions in this group.[38] The current work informs the risk-benefit analysis undertaken as part of antipsychotic drug prescribing in people with ID and reinforces the need for regular and effective medication review, which must include assessment of possible movement side-effects.

For peer review only

References

1. Marsden C, Jenner P. The pathophysiology of extrapyramidal side-effects of neuroleptic drugs. *Psychological medicine* 1980; **10**: 55-72.

2. Leucht S, Wahlbeck K, Hamann J, Kissling W. New generation antipsychotics versus low-potency conventional antipsychotics: a systematic review and meta-analysis. *The Lancet* 2003; **361**: 1581-9.

3. Cooper S-A, Smiley E, Morrison J, Williamson A, Allan L. Mental ill-health in adults with intellectual disabilities: prevalence and associated factors. *The British Journal of Psychiatry* 2007; **190**: 27-35.

4. Sheehan R, Hassiotis A, Walters K, Osborn D, Strydom A, Horsfall L. Mental illness, challenging behaviour, and psychotropic drug prescribing in people with intellectual disability: UK population based cohort study. *BMJ* 2015; **351**: h4326.

5. Deb S, Sohanpal S, Soni R, Lentre L, Unwin G. The effectiveness of antipsychotic medication in the management of behaviour problems in adults with intellectual disabilities. *Journal of Intellectual Disability Research* 2007; **51**: 766-77.

6. Arnold LE. Clinical pharmacological issues in treating psychiatric disorders of patients with mental retardation. *Annals of clinical psychiatry* 1993; **5**: 189-97.

7. Cunningham-Owens DG. Special populations. A guide to extrapyramidal side-effects of antipsychotic drugs. 2nd ed. Cambridge, UK: Cambridge University Press; 2014.

8. Blak B, Thompson M, Dattani H, Bourke A. Generalisability of The Health Improvement Network (THIN) database: demographics, chronic disease prevalence and mortality rates. *Journal of Innovation in Health Informatics* 2011; **19**: 251-5.

9. Bourke A, Dattani H, Robinson M. Feasibility study and methodology to create a quality-evaluated database of primary care data. *Journal of Innovation in Health Informatics* 2004; **12**: 171-7.

10. Booth N. What are the Read codes? *Health libraries review* 1994; **11**: 177-82.

11. Maguire A, Blak BT, Thompson M. The importance of defining periods of complete mortality reporting for research using automated data from primary care. *Pharmacoepidemiology and drug safety* 2009; **18**: 76-83.

12. Horsfall L, Walters K, Petersen I. Identifying periods of acceptable computer usage in primary care research databases. *Pharmacoepidemiology and drug safety* 2013; **22**: 64-9.

13. Davé S, Petersen I. Creating medical and drug code lists to identify cases in primary care databases. *Pharmacoepidemiology and drug safety* 2009; **18**: 704-7.

For peer review only

14. Lewis JD, Bilker WB, Weinstein RB, Strom BL. The relationship between time since registration and measured incidence rates in the General Practice Research Database. *Pharmacoepidemiology and drug safety* 2005; **14**: 443-51.

15. Townsend P, Phillimore P, Beattie A. Health and deprivation: inequality and the North. Routledge; 1988.

16. Marston L, Nazareth I, Petersen I, Walters K, Osborn DP. Prescribing of antipsychotics in UK primary care: a cohort study. *BMJ Open* 2014; **4**: e006135.

17. Frighi V, Stephenson MT, Morovat A, et al. Safety of antipsychotics in people with intellectual disability. *The British Journal of Psychiatry* 2011; **199**: 289-95.

18. Gagiano C, Read S, Thorpe L, Eerdekens M, Van Hove I. Short-and long-term efficacy and safety of risperidone in adults with disruptive behavior disorders. *Psychopharmacology* 2005; **179**: 629-36.

19. Leucht S, Cipriani A, Spineli L, et al. Comparative efficacy and tolerability of 15 antipsychotic drugs in schizophrenia: a multiple-treatments meta-analysis. *The Lancet* 2013; **382**: 951-62.

20. Peluso MJ, Lewis SW, Barnes TR, Jones PB. Extrapyramidal motor side-effects of first-and second-generation antipsychotic drugs. *The British Journal of Psychiatry* 2012; **200**: 387-92.

21. Scheifes A, Walraven S, Stolker JJ, et al. Movement Disorders in Adults With Intellectual Disability and Behavioral Problems Associated With Use of Antipsychotics. *Journal of clinical psychopharmacology* 2016; **36**: 308-13.

22. de Kuijper G, Mulder H, Evenhuis H, Scholte F, Visser F, Hoekstra PJ. Determinants of physical health parameters in individuals with intellectual disability who use long-term antipsychotics. *Research in developmental disabilities* 2013; **34**: 2799-809.

23. Matson JL, Fodstad JC, Neal D, Dempsey T, Rivet TT. Risk factors for tardive dyskinesia in adults with intellectual disability, comorbid psychopathology, and long-term psychotropic use. *Research in developmental disabilities* 2010; **31**: 108-16.

24. Rogers D, Karki C, Bartlett C, Pocock P. The motor disorders of mental handicap. An overlap with the motor disorders of severe psychiatric illness. *The British Journal of Psychiatry* 1991; **158**: 97-102.

25. Guy W. Abnormal involuntary movement scale. ECDEU Assessment Manual for Psychopharmacology. Washington, DC: US Department of Health, Education and Welfare; 1976.

26. Barnes TR. A rating scale for drug-induced akathisia. *The British Journal of Psychiatry* 1989; **154**: 672-6.

27. Simpson G, Angus J. A rating scale for extrapyramidal side effects. *Acta Psychiatrica Scandinavica* 1970; **45**: 11-9.

For peer review only

28. Viejo L, Morales V, Punal P, Perez J, Sancho R. Risk factors in neuroleptic malignant syndrome. A case–control study. *Acta Psychiatrica Scandinavica* 2003; **107**: 45-9.

29. Shalev A, Munitz H. The neuroleptic malignant syndrome: agent and host interaction. *Acta Psychiatrica Scandinavica* 1986; **73**: 337-47.

30. Boyd RD. Neuroleptic malignant syndrome and mental retardation: review and analysis of 29 cases. *American journal on mental retardation* 1993; **98**: 143-55.

31. Verdoux H, Tournier M, Begaud B. Antipsychotic prescribing trends: a review of pharmaco-epidemiological studies. *Acta Psychiatrica Scandinavica* 2010; **121**: 4-10.

32. Haddad PM, Dursun SM. Neurological complications of psychiatric drugs: clinical features and management. *Human Psychopharmacology: Clinical and Experimental* 2008; **23**: S15-S26.

33. Crossley R, Withers P. Antipsychotic medication and people with intellectual disabilities: their knowledge and experiences. *Journal of Applied Research in Intellectual Disabilities* 2009; **22**: 77-86.

34. Lennox N, Diggens J, Ugoni A. The general practice care of people with intellectual disability: barriers and solutions. *Journal of Intellectual Disability Research* 1997; **41**: 380-90.

35. Schouten HJ, Knol W, Egberts TC, Schobben AF, Jansen PA, van Marum RJ. Quality of life of elderly patients with antipsychotic-induced parkinsonism: A cross-sectional study. *Journal of the American Medical Directors Association* 2012; **13**: 82. e1-. e5.

36. Scheifes A, Walraven S, Stolker JJ, Nijman HL, Egberts TC, Heerdink ER. Adverse events and the relation with quality of life in adults with intellectual disability and challenging behaviour using psychotropic drugs. *Research in developmental disabilities* 2016; **49**: 13-21.

37. Paton C, Bhatti S, Purandare K, Roy A, Barnes TRE. Quality of prescribing of antipsychotic medication for people with intellectual disability under the care of UK mental health services: a cross-sectional audit of clinical practice. *BMJ Open* 2016; **6**: e013116.

38. Sheehan R, Hassiotis A. Reduction or discontinuation of antipsychotics for challenging behaviour in adults with intellectual disability: a systematic review. *The Lancet Psychiatry* 2016; **4**: 238-56.

For peer review only

TABLE 1 – Cohort characteristics

	ID cohort	Non-ID cohort
Total number (%)	9,039 (21)	34,242 (79)
Male, *n* (%)	5,279 (58)	18,825 (55)
Average age, *yrs* (SD)	42 (16)	44 (16)
Townsend score, *n* (%)		
1	1,403 (16)	4,860 (14)
2	1,752 (19)	5,119 (15)
3	1,948 (22)	6,481 (19)
4	1,942 (22)	8,189 (24)
5	1,563 (17)	7,911 (23)
Missing	417 (5)	1,619 (5)
History of antipsychotic use at cohort entry, *n* (%)	6,684 (74)	16,227 (47)
History of movement disorder at cohort entry, *n* (%)	2,192 (24)	4,946 (14)
History of movement disorder without antipsychotic use at cohort entry, *n* (%)	136/2,355 (6)	1,038/18,015 (6)
History of movement disorder and antipsychotic use at cohort entry, *n* (%)	2,056/6,684 (31)	3,908/16,227 (24)
Total person-years between first and last antipsychotic prescription	44,696	104,014
Median years between first and last prescription (IQR)	3.5 (1.2 to 7.9)	1.3 (0.19 to 4.4)
Median years on treatment between first and last prescription (IQR)	2.6 (0.76 to 6.3)	0.67 (0.15 to 2.5)
Average daily dose, *CLZE* (SD)	135 (156)	139 (146)

CLZE, Chlorpromazine equivalents

For peer review only

TABLE 2 – frequency of antipsychotic drugs prescribed in the intellectually disabled and non-intellectually disabled cohorts

**Antipsychotic drug**	**ID cohort**				**Non-ID cohort**			
	Number	Percentage	Average daily dose, mg	Median treatment duration, yrs	Number	Percentage	Average daily dose, mg	Median treatment duration, yrs
Risperidone	4,013	28.5	1.9	1.81	7,426	14.7	2.2	0.47
Olanzapine	2,086	14.8	8.2	1.62	10,246	20.3	8.5	0.67
Chlorpromazine	1,770	12.6	78.5	1.12	5,274	10.5	64.8	0.20
Quetiapine	1,295	9.2	154.1	0.93	7,693	15.2	152.6	0.72
Haloperidol	1,231	8.7	4.9	0.68	3,755	7.5	3.2	0.14
Thioridazine	838	6.0	77.9	0.82	2,672	5.3	49.8	0.20
Aripiprazole	661	4.7	10.5	0.74	2,638	5.2	11.8	0.54
Trifluoperazine	456	3.2	6.5	1.06	3,403	6.8	4.5	0.19
Zuclopenthixol	429	3.1	18.7	2.17	340	0.7	20.0	0.45
Amisulpride	327	2.3	295.0	0.94	1,687	3.4	290.3	0.73
Promazine	276	2.0	58.4	0.33	1,688	3.4	58.6	0.16
Sulpiride	276	2.0	437.8	1.97	1,199	2.4	435.6	0.79
Other*	980	7.0	-	-	5,230	10.4	-	-

*Other antipsychotic drugs prescribed to <1% of ID cohort each

For peer review only

TABLE 3 – Incidence rates and adjusted incident rate ratios for movement side-effects in people with and without intellectual disability prescribed antipsychotic drugs

	ID cohort			Non-ID cohort			Comparison	
**Variable**	No of events during follow-up	No of Person-Years (X10,000)	Incidence per 10,000 Person-Years (95%CI)	No of events during follow-up	No of Person-Years (X10,000)	Incidence per 10,000 Person-Years (95%CI)	Incidence Rate Ratio* (95%CI)	p-value
**Any movement disorder (defined by Read code or antimuscarinic prescription)**	**743**	**2.7**	**275 (256 to 296)**	**1750**	**7.0**	**248 (237 to 260)**	**1.30 (1.18 to 1.42)**	**<0.001**
**Any movement disorder (defined by Read code)**	**446**	**4.4**	**111 (101 to 122)**	**952**	**9.4**	**101 (95 to 108)**	**1.30 (1.16 to 1.47)**	**<0.001**
**Any movement disorder (defined by antimuscarinic prescription)**	**564**	**2.9**	**196 (180 to 212)**	**1299**	**7.6**	**172 (163 to 181)**	**1.29 (1.16 to 1.44)**	**<0.001**
Acute dystonia	60	4.4	14 (11 to 18)	161	10.2	16 (14 to 19)	1.00 (0.73 to 1.37)	0.99
**Akathisia**	**80**	**4.5**	**18 (15 to 23)**	**112**	**10.3**	**11 (9 to 13)**	**2.29 (1.69 to 3.12)**	**<0.001**
**Parkinsonism**	**270**	**4.4**	**64 (57 to 72)**	**592**	**9.9**	**60 (55 to 65)**	**1.20 (1.03 to 1.39)**	**0.02**
Tardive dyskinesia	61	4.0	14 (11 to 18)	123	10.3	12 (10 to 14)	1.27 (0.91 to 1.75)	0.16
**Neuroleptic malignant syndrome**	**11**	**4.4**	**3 (1 to 5)**	**12**	**10.4**	**1 (1-2)**	**3.03 (1.26 to 7.30)**	**0.013**
Other movement disorder	43	4.2	10 (7 to 13)	94	10.3	9 (7-11)	1.26 (0.86 to 1.85)	0.23

*Adjusted for sex, social deprivation score, time period, history of antipsychotic drug use, average daily dose, days on treatment

For peer review only

TABLE 4 – Sensitivity analysis with incidence rates and adjusted incident rate ratios for movement side-effects in people with and without intellectual disability prescribed first- and second-generation antipsychotic drugs

	**ID cohort**			**Non-ID cohort**			**Comparison**	
**Antipsychotic class**	No of events during follow-up	No of Person-Years (X10,000)	Incidence per 10,000 Person-Years (95%CI)	No of events during follow-up	No of Person-Years (X10,000)	Incidence per 10,000 Person-Years (95%CI)	Incidence Rate Ratio* (95%CI)	p-value
**First generation****	**247**	**0.8**	**320 (283 to 362)**	**569**	**1.9**	**293 (270 to 318)**	**1.36 (1.16 to 1.60)**	**<0.001**
**Second generation****	**378**	**1.6**	**241 (218 to 267)**	**948**	**4.3**	**219 (206 to 233)**	**1.43 (1.26 to 1.62)**	**<0.001**
**Risperidone****	**124**	**0.6**	**196 (164 to 233)**	**96**	**0.5**	**182 (149 to 223)**	**1.55 (1.15 to 2.08)**	**0.004**

*Adjusted for sex, social deprivation score, time period, history of antipsychotic drug use, average daily dose, days on treatment

** Restricted to periods when people were exclusively prescribed first- or second-generation antipsychotic drugs or risperidone

For peer review only

Figure legends

Figure 1a and figure 1b

Time trends in crude incidence rates of a) movement side-effect defined by Read code and b) antimuscarinic drug prescription in people with and without intellectual disability prescribed antipsychotic drugs

For peer review only

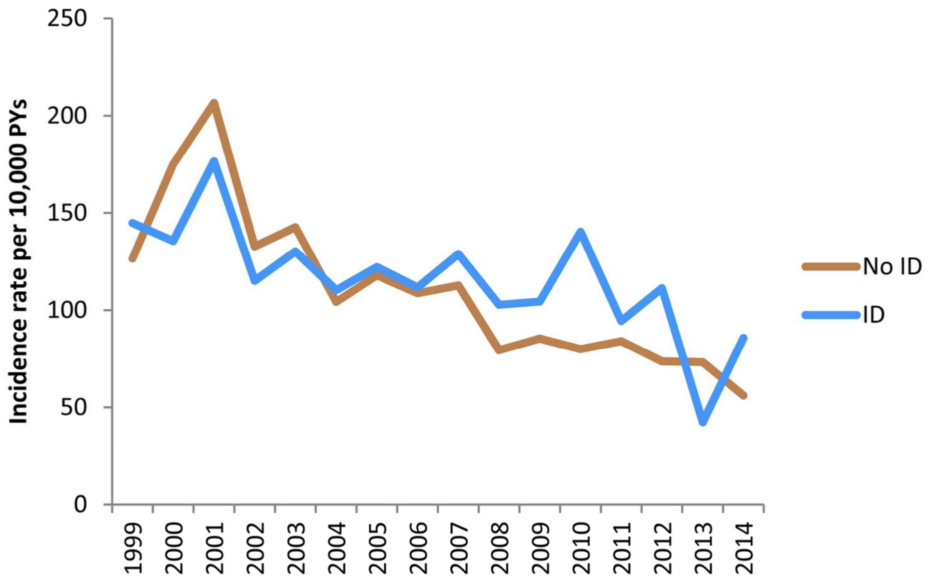

Caption : Time trends in crude incidence rates of a) movement side-effect defined by Read code and b) antimuscarinic drug prescription in people with and without intellectual disability prescribed antipsychotic drugs

129x88mm (300 x 300 DPI)

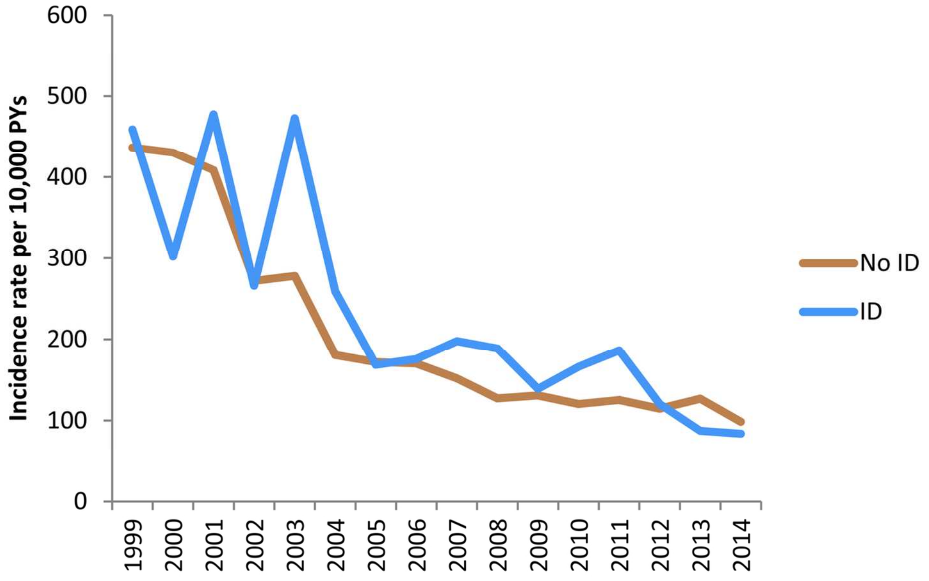

Caption : Time trends in crude incidence rates of a) movement side-effect defined by Read code and b) antimuscarinic drug prescription in people with and without intellectual disability prescribed antipsychotic drugs

131x90mm (300 x 300 DPI)

Movement side-effects of antipsychotic drugs in adults with and without intellectual disability: UK population-based cohort study

Sheehan R, Horsfall L, Strydom A, Osborn D, Walters K, Hassiotis A

Antipsychotic movement side-effects by type

Acute dystonia

16A3.00	Wry neck/torticollis
16A3.11	Torticollis - symptom
1B25.00	Has "spasms"
1B25.11	Spasms - symptom
1B35.00	Attacks of rigidity
1B36.00	Trismus present
22B2.00	O/E - carpopedal spasm
2942000	Trismus
2974.00	O/E - spasm/tic
2974.11	O/E - spasm
7Q04000	Torsion dystonias other involuntary movements drugs band 1
F137.00	Symptomatic torsion dystonia
F137200	Drug-induced dystonia
F137y00	Other specified symptomatic torsion dystonia
F137z00	Symptomatic torsion dystonia NOS
F138.00	Fragments of torsion dystonia
F138000	Blepharospasm
F138200	Spasmodic torticollis
F13X.00	Dystonia, unspecified
F4Jy911	Oculogyric crisis
Fyu2400	[X]Other dystonia
Fyu2A00	[X]Dystonia, unspecified
N135.00	Torticollis unspecified
N135000	Intermittent torticollis
N135z00	Torticollis NOS
R010200	[D]Spasms NOS
R010600	[D] Trismus
R017000	[D]Carpopedal spasm

Pseudo-Parkinsonism

1B22.00	Has a tremor
1B22.11	Tremor symptom
1B22.12	Shaking
1B23.00	Trembles
1B23.11	Trembles - symptom
294..11	O/E - rigid muscle
2942.00	O/E - muscle tone hypertonic
2944.00	O/E - muscle rigid - cogwheel
2944.11	O/E - cog wheel rigidity

For peer review only

Movement side-effects of antipsychotic drugs in adults with and without intellectual disability: UK population-based cohort study

Sheehan R, Horsfall L, Strydom A, Osborn D, Walters K, Hassiotis A

297A.00	O/E - Parkinsonian tremor
2987.00	O/E -Parkinson flexion posture
2987.11	O/E - Parkinson posture
2994.00	O/E-festination-Parkinson gait
2994.11	O/E - Parkinson gait
F121.00	Parkinsonism secondary to drugs
F121.11	Drug induced parkinsonism
F12W.00	Secondary parkinsonism due to other external agents
F12X.00	Secondary parkinsonism, unspecified
F131200	Drug-induced tremor
Fyu2000	[X]Other drug-induced secondary parkinsonism
Fyu2100	[X]Other secondary parkinsonism
Fyu2900	[X]Secondary parkinsonism, unspecified
R010300	[D]Tremor NOS
Fyu2B00	[X]Secondary parkinsonism due to other external agents

Akathisia

1B1O.00	Restless
1P04.00	C/O - akathisia

Tardive dyskinesia

F138100	Orofacial dyskinesia
F138111	Tardive dyskinesia
297..00	O/E - involuntary movements
297Z.00	O/E - involuntary movement NOS
1B2..00	Involuntary movement symptom
1B2Z.00	Involuntary movemt.symptom NOS
R010.00	[D]Abnormal involuntary movements
R010z00	[D]Abnormal involuntary movement NOS
Ryu3000	[X]Other and unspecified abnormal involuntary movements
1B2Z.00	Involuntary movemt.symptom NOS
1B2..00	Involuntary movement symptom

Other/misc.

Fyu2700	[X]Other specified extrapyramidal and movement disorders
R013.11	[D]Dyskinesia
F13z.00	Other/unspecified extrapyramidal/abnormal movement disorders
F13z000	Unspecified extrapyramidal disease
F13zz00	Extrapyramidal disease and abnormal movement disorder NOS
Fyu2.00	[X]Extrapyramidal and movement disorders
29M..00	Extrapyramidal movements
F13..00	Other extrapyramidal disease and abnormal movement disorders

For peer review only

Movement side-effects of antipsychotic drugs in adults with and without intellectual disability: UK population-based cohort study

Sheehan R, Horsfall L, Strydom A, Osborn D, Walters K, Hassiotis A

F13..11	Extrapyramidal disease excluding Parkinson's disease
ZS42500	Extrapyramidal dysarthria

Neuroleptic malignant syndrome

F122.00	Malignant neuroleptic syndrome

For peer review only

STROBE Statement—checklist of items that should be included in reports of observational studies

Movement side-effects of antipsychotic drugs in adults with intellectual disability

	Item No.	Recommendation	Page No.	Relevant text from manuscript
**Title and abstract**	1	(*a*) Indicate the study's design with a commonly used term in the title or the abstract	1	Cohort study in title
		(*b*) Provide in the abstract an informative and balanced summary of what was done and what was found	2	
**Introduction**				
Background/rationale	2	Explain the scientific background and rationale for the investigation being reported	6	"People with ID are often considered to be at greater risk of antipsychotic drug-induced movement side-effects than people without ID but no studies directly compare rates"
Objectives	3	State specific objectives, including any prespecified hypotheses	7	"To compare the incidence of recorded movement side-effects in adults with and without ID who were prescribed antipsychotic drugs".
**Methods**				
Study design	4	Present key elements of study design early in the paper	7-9	Data source, study cohort, covariates subheadings
Setting	5	Describe the setting, locations, and relevant dates, including periods of recruitment, exposure, follow-up, and data collection	7-8	Study cohort subheading
Participants	6	(*a*) *Cohort study*—Give the eligibility criteria, and the sources and methods of selection of participants. Describe methods of follow-up<br>*Case-control study*—Give the eligibility criteria, and the sources and methods of case ascertainment and control selection. Give the rationale for the choice of cases and controls<br>*Cross-sectional study*—Give the eligibility criteria, and the sources and methods of selection of	7-8	Study cohort subheading "all adults with ID…were extracted"

For peer review only - http://bmjopen.bmj.com/site/about/guidelines.xhtml

		participants		
		(*b*) *Cohort study*—For matched studies, give matching criteria and number of exposed and unexposed<br>*Case-control study*—For matched studies, give matching criteria and the number of controls per case	8	"A comparison cohort…was extracted using stratified sampling within each GP practice with frequency matching…"
Variables	7	Clearly define all outcomes, exposures, predictors, potential confounders, and effect modifiers. Give diagnostic criteria, if applicable	7-9	Read code lists described<br>Covariates described
Data sources/ measurement	8*	For each variable of interest, give sources of data and details of methods of assessment (measurement). Describe comparability of assessment methods if there is more than one group	7	Data source subheading
Bias	9	Describe any efforts to address potential sources of bias	8	"Comparison cohort matched to ensure similar population level characteristics across the two groups"
Study size	10	Explain how the study size was arrived at	7	"All adults with recorded ID"

Continued on next page

For peer review only - http://bmjopen.bmj.com/site/about/guidelines.xhtml

Quantitative variables	11	Explain how quantitative variables were handled in the analyses. If applicable, describe which groupings were chosen and why	9	Statistical analysis subheading
Statistical methods	12	(*a*) Describe all statistical methods, including those used to control for confounding	9	"Multivariable mixed Poisson regression was used…"
		(*b*) Describe any methods used to examine subgroups and interactions		
		(*c*) Explain how missing data were addressed	9	"It was not possible to estimate the daily dose for 5% of prescriptions and these were excluded from the study".
		(*d*) *Cohort study*—If applicable, explain how loss to follow-up was addressed<br>*Case-control study*—If applicable, explain how matching of cases and controls was addressed<br>*Cross-sectional study*—If applicable, describe analytical methods taking account of sampling strategy	NA	
		(*e*) Describe any sensitivity analyses	9	"We conducted a sensitivity analysis…"
**Results**				
Participants	13*	(a) Report numbers of individuals at each stage of study—eg numbers potentially eligible, examined for eligibility, confirmed eligible, included in the study, completing follow-up, and analysed	NA	
		(b) Give reasons for non-participation at each stage	NA	
		(c) Consider use of a flow diagram	NA	
Descriptive data	14*	(a) Give characteristics of study participants (eg demographic, clinical, social) and information on exposures and potential confounders	10<br>Table 1	
		(b) Indicate number of participants with missing data for each variable of interest		
		(c) *Cohort study*—Summarise follow-up time (eg, average and total amount)	Table 1	Total person years between first and last prescription, median years between first and last prescription
Outcome data	15*	*Cohort study*—Report numbers of outcome events or summary measures over time	10<br>Table 3	"The overall incidence of recorded movement side effects…"
		Case-control study—Report numbers in each exposure category, or summary measures of exposure	NA	
		Cross-sectional study—Report numbers of outcome events or summary measures	NA	
Main results	16	(*a*) Give unadjusted estimates and, if applicable, confounder-adjusted estimates and their precision	10	

For peer review only

For peer review only - http://bmjopen.bmj.com/site/about/guidelines.xhtml

	(eg, 95% confidence interval). Make clear which confounders were adjusted for and why they were included	Table 2
	(*b*) Report category boundaries when continuous variables were categorized	NA
	(*c*) If relevant, consider translating estimates of relative risk into absolute risk for a meaningful time period	

Continued on next page

For peer review only

For peer review only - http://bmjopen.bmj.com/site/about/guidelines.xhtml

Other analyses	17	Report other analyses done—eg analyses of subgroups and interactions, and sensitivity analyses	11<br>Table 4	"Analysis restricted to periods when people were exclusively prescribed first- or second-generation antipsychotic drugs did not change the results".
**Discussion**				
Key results	18	Summarise key results with reference to study objectives	11	"Our data suggest that people with ID are indeed more likely to experience movement side-effects of antipsychotic drugs".
Limitations	19	Discuss limitations of the study, taking into account sources of potential bias or imprecision. Discuss both direction and magnitude of any potential bias	14-15	
Interpretation	20	Give a cautious overall interpretation of results considering objectives, limitations, multiplicity of analyses, results from similar studies, and other relevant evidence	15	"People with ID appear more susceptible to movement side-effects of antipsychotic drugs than people without ID".
Generalisability	21	Discuss the generalisability (external validity) of the study results		
**Other information**				
Funding	22	Give the source of funding and the role of the funders for the present study and, if applicable, for the original study on which the present article is based	5	"The Baily Thomas Charitable Fund and the National Institute for Health Research"

*Give information separately for cases and controls in case-control studies and, if applicable, for exposed and unexposed groups in cohort and cross-sectional studies.

Note: An Explanation and Elaboration article discusses each checklist item and gives methodological background and published examples of transparent reporting. The STROBE checklist is best used in conjunction with this article (freely available on the Web sites of PLoS Medicine at http://www.plosmedicine.org/, Annals of Internal Medicine at http://www.annals.org/, and Epidemiology at http://www.epidem.com/). Information on the STROBE Initiative is available at www.strobe-statement.org.

For peer review only - http://bmjopen.bmj.com/site/about/guidelines.xhtml
